# Simulated Interventions to Ameliorate Age-Related Bone Loss Indicate the Importance of Timing

**DOI:** 10.3389/fendo.2016.00061

**Published:** 2016-06-13

**Authors:** Carole J. Proctor, Alison Gartland

**Affiliations:** ^1^MRC/Arthritis Research UK Centre for Musculoskeletal Ageing (CIMA), Newcastle University, Newcastle upon Tyne, UK; ^2^Musculoskeletal Research Group, Institute of Cellular Medicine, Medical School, Newcastle University, Newcastle upon Tyne, UK; ^3^Department of Oncology and Metabolism, University of Sheffield Medical School, Sheffield, UK

**Keywords:** bone remodeling, circadian cycle, cell signaling, mechanical loading, parathyroid hormone, stochastic simulation

## Abstract

Bone remodeling is the continuous process of bone resorption by osteoclasts and bone formation by osteoblasts, in order to maintain homeostasis. The activity of osteoclasts and osteoblasts is regulated by a network of signaling pathways, including Wnt, parathyroid hormone (PTH), RANK ligand/osteoprotegrin, and TGF-β, in response to stimuli, such as mechanical loading. During aging there is a gradual loss of bone mass due to dysregulation of signaling pathways. This may be due to a decline in physical activity with age and/or changes in hormones and other signaling molecules. In particular, hormones, such as PTH, have a circadian rhythm, which may be disrupted in aging. Due to the complexity of the molecular and cellular networks involved in bone remodeling, several mathematical models have been proposed to aid understanding of the processes involved. However, to date, there are no models, which explicitly consider the effects of mechanical loading, the circadian rhythm of PTH, and the dynamics of signaling molecules on bone remodeling. Therefore, we have constructed a network model of the system using a modular approach, which will allow further modifications as required in future research. The model was used to simulate the effects of mechanical loading and also the effects of different interventions, such as continuous or intermittent administration of PTH. Our model predicts that the absence of regular mechanical loading and/or an impaired PTH circadian rhythm leads to a gradual decrease in bone mass over time, which can be restored by simulated interventions and that the effectiveness of some interventions may depend on their timing.

## Introduction

Bone remodeling is the continuous process of bone resorption and formation in order to repair micro-fractures and maintain mineral homeostasis. Osteoclasts and osteoblasts work together in “basic multicellular units” (BMUs) to resorb and form new bone, respectively. An imbalance in bone remodeling occurs in disease, such as Paget’s disease or cancer, and loss of homeostasis may occur in aging. In particular, loss of bone mass occurs with age and may lead to osteoporosis. This may be due to a decline in the number of osteoblasts with age. Osteoblasts are derived from mesenchymal stem cells (MSCs) (1) and are induced to differentiate by activation of the Wnt signaling pathway and by growth factors, e.g., BMPs and TGF-β. After osteoblasts have carried out bone formation, they either mature into osteocytes, which are embedded in the new bone matrix, form lining cells, or undergo apoptosis.

Osteoclasts are derived from hematopoietic stem cells (HSCs) (2). Differentiation of HSCs into progenitor cells requires macrophage colony-stimulating factor (MCSF) (3), which is secreted by osteoblasts and MSCs (3). Osteoclast progenitor cells express receptor activator of NFκB (RANK) receptors and require the RANK ligand (RANKL) for further differentiation into mature osteoclasts (Ocl_m) (4). After osteoclasts have carried out the resorption process, they undergo apoptosis.

Osteocytes are buried deep in the bone matrix and make up >90% of cells in bone. Although it was once thought that these cells are quiescent, it is now known that they have an important role in maintenance of bone homeostasis, as they respond to mechanical stimuli (by receiving signals from integrins) and secrete signaling molecules. In the absence of mechanical forces, osteocytes may undergo apoptosis (5). This may be due to a decrease in levels of nitric oxide (6) or prostaglandin E_2_ (PGE_2_) (7).

Many signaling pathways are important in bone remodeling. In particular, the RANK–RANKL–OPG pathway plays a major role in bone remodeling (8, 9). Osteoprotegrin (OPG) is a decoy ligand, which binds to RANKL, thus preventing RANKL binding to RANK. Therefore, the RANKL/OPG ratio is very important in maintaining bone homeostasis. MSCs, osteoblasts (mature and precursor cells), and osteocytes secrete RANKL. It is thought that MSCs only express low levels of RANKL. The general consensus was that osteoblasts are the main source of RANKL, but there has recently been a paradigm shift, and osteocytes are now considered to be important [e.g., Ref. (10)]. Osteoblasts also secrete OPG. It has been suggested that osteoblast precursor cells (Ob_p) secrete high levels of RANKL and low levels of OPG, whereas the reverse is true for mature osteoblasts (Ob_m) (11), so that the RANKL/OPG ratio changes during osteoblast differentiation. This would mean that osteoclastogenesis is promoted during early differentiation of osteoblasts, but inhibited at later stages.

An important pathway for osteoblastogenesis is TGF-β signaling. TGF-β is required for differentiation of MSCs into osteoblast precursors, but inhibits later stages of the differentiation process (12). TGF-β is produced and secreted by osteoblasts and is abundantly present in the bone matrix in a latent form. It is released from the bone matrix by osteoclasts during the process of bone resorption (13).

Another important pathway in bone remodeling is canonical Wnt signaling. Wnt is known to promote osteoblast proliferation and differentiation (14). Osteocytes secrete sclerostin, which inhibits Wnt signaling, especially during unloading when they are in a quiescent state (15, 16). It has been shown that activation of Wnt prevents osteocyte apoptosis (14), suggesting that apoptosis of osteocytes occurs when they are inactive during unloading. Parathyroid hormone (PTH) also leads to activation of osteocytes and may compensate for a lack of mechanical stimulation [reviewed in Ref. (17)].

Parathyroid hormone receptors are found on osteoblasts and osteocytes. Signaling *via* PTH leads to an increase in the RANKL/OPG ratio due to inhibition of OPG secretion and an increase in RANKL secretion from osteoblasts (18), leading to more bone resorption. On the other hand, PTH can increase bone formation by a number of mechansims. For example, it is known to increase Wnt signaling by activating osteocytes and so lowering levels of the Wnt inhibitor, sclerostin (19). PTH also increases Wnt signaling by changing the expression of Wnt signaling components *via* the cAMP-PKA pathway (20). Furthermore, PTH inhibits osteoblast apoptosis, as, when, PTH binds to receptors on osteoblasts, a signaling cascade involving phosphorylation of CREB is activated (21). Phosphorylated CREB binds to Runx2, which leads to the nuclear translocation and transcriptional activity of Runx2, a key transcription factor in bone formation. One of the genes regulated by Runx2 is Bcl2, which binds to the proapoptotic protein Bax to inhibit apoptosis of osteoblasts. PTH also enhances the degradation of Runx2, which provides a negative feedback loop to terminate transcription of Runx2 genes. PTH has a very short half-life of only a few minutes (22), so that it is normally kept at low basal levels. It has been suggested that PTH is normally present below the threshold required for signaling and that other signaling pathways are required, e.g., purinergic signaling (23). Previous experiments have shown that continuous administration of PTH leads to bone loss, whereas intermittent PTH results in bone formation (24–26). Importantly, PTH levels have a circadian rhythm in humans, and this rhythm is disturbed in postmenopausal women. Joseph et al. (27) showed that elderly men and premenopausal women have two peaks of PTH per day (a lower peak in late afternoon, followed by a higher nocturnal peak), whereas postmenopausal women have a higher sustained increase in nocturnal PTH levels without the earlier peak.

Several mathematical models of bone remodeling have been published (28–34). Many of these models use ordinary differential equations to model the differentiation and dynamics of osteoblast and osteoclast cells. They include the effects of chemical species, such as OPG, RANKL, PTH, and TGF-β, but do not follow the dynamics of these species, instead representing them as parameters in reaction rates. Graham et al. (29) extended previous models to include the role of osteocytes, sclerostin, and the contribution of osteocytes to RANKL secretion. Currently, there are few models that include the role of mechanical loading on bone remodeling. Scheiner et al. (35) extended previous models (32, 33) and were the first to include mechanoregulatory feedback mechanisms by combining bone cell population kinetics with multiscale bone mechanics. Pivonka et al. (36) developed this further to include the bone microstructure in which they examined the influence of bone surface availability. However, to date, we are not aware of any mathematical model that explicitly examines the effect of changes in the circadian rhythm of PTH on the different signaling pathways involved in bone remodeling or the interplay between periodical mechanical loading and the PTH cycle on the system. The aim of this study was to construct an integrative computational model of bone remodeling in order to examine the effects of loading/unloading, the PTH circadian rhythm, and their synergistic effects, if any, on bone homeostasis. After establishing which conditions lead to loss of bone mass, we used the model to simulate different interventions, such as RANKL inhibitors, sclerostin inhibitors, and bisphosphonates, in order to try and restore bone homeostasis. In particular, since both mechanical loading and PTH have periodical effects over limited time periods, we used the model to investigate the question of whether the actual timing of interventions, such as administration of PTH or bouts of physical exercise, affect the dynamics of bone remodeling.

We also examined the effects of different PTH treatments in order to validate our model against experimental data, which indicate opposing effects of continuous versus intermittent PTH administration [e.g., Ref. (37, 38)]. The reasons for this are not fully understood and are likely to be due to a combination of mechanisms (39). For example, the increase in bone mass by intermittent PTH may be due to downregulation of sclerostin (40), leading to an increase in osteoblast number and more bone formation. Previous mathematical models have also examined the opposing effects of PTH, using different model assumptions. For example, Kroll assumed that PTH enhanced the differentiation of osteoblast progenitor cells (Ob_pro), but inhibited the differentiation of osteoblasts precursor cells into Ob_m (34). On the other hand, Lemaire et al. assumed that PTH binds to receptors on Ob_m, whereby it stimulates production of RANKL, but inhibits production of OPG (31). We based our model on some of the assumptions of earlier models, but we also included the dynamics of chemical species, including OPG, RANKL, PTH, Runx2, Sclerostin, TGF-β, and Wnt. In addition, we included explicit mechanisms to mimic loading and the circadian rhythm of PTH in our model. We used a modular approach for the model construction using the modeling standard, Systems Biology Markup Language (SBML) (41), which will allow for modifications and further extensions, as required.

## Materials and Methods

The model was based on a number of assumptions supported by the literature, but to avoid too much complexity at this stage of the model development, detail of some of the processes has been omitted. For example, we modeled mechanical loading by including a dummy species “LOAD,” which could be turned on and off during the simulation and simply assumed that osteocytes are activated in the presence of LOAD. This was represented by the reaction Ocy_I + LOAD → Ocy_A + LOAD, where Ocy_I and Ocy_A are the pools of inactive and active osteocytes, respectively. For most of the reactions, we used mass action kinetics. We also used Hill kinetics for some of the second-order reactions. For example, in reactions that represent binding of ligands to receptors, we assumed that the reaction would be limited by the number of receptors. This prevents activation of signaling pathways when ligands are present at low levels with an approximately linear increase in the rate for intermediate levels, which then reaches a maximum rate to represent saturation of the ligand. The model assumptions are given below.

The models were encoded in SBML (41), a modeling standard for biochemical networks, using the Python tool SBML shorthand (42). Model diagrams were constructed in CellDesigner (43) using the Systems Biology Graphical Notation (SBGN) (44) and deterministic simulations were carried out in COPASI (45). Stochastic simulations were carried out on a cluster with our own simulator (gillespie2) (46), which is based on the Gillespie algorithm (47). The SBML code was deposited in the Biomodels database and assigned the identifier MODEL1602290000 (48). All graphs were constructed using the R package ggplot2 (49).

### Model Assumptions

The model was based on a number of assumptions that are given below. Each component (cell type or protein) in the model has a unique species ID and is shown in brackets if it differs from the component name.

Hematopoietic progenitor cells differentiate into osteoclast precursor cells (Ocl_p). This requires MCSF (3).

Osteoclast precursor cells differentiate into Ocl_m. This requires RANKL binding to Ocl_p (4). Precursor cells may undergo apoptosis.

Mature osteoclasts degrade bone, activate TGF-β (Tgfb_A), and undergo apoptosis.

Mesenchymal stem cells differentiate into Ob_pro. This requires active Wnt (Wnt_A) (50). MSCs also secrete MCSF (3) and RANKL (51).

Osteoblast progenitor cells differentiate into Ob_p. This requires active TGF-β (12). Ob_pro also secretes MCSF and RANKL (11).

Osteoblast precursor cells differentiate into Ob_m. Ob_p may secrete MCSF, OPG, and RANKL (11). Tgfb_A and PTH reversibly bind to Ob_p to inhibit differentiation of Ob_p into mature cells (12). Ob_m form new bone, secrete MCSF, OPG, and RANKL (11), mature into inactive osteocytes (Ocy_I), or undergo apoptosis. Maturation of Ob_m into osteocytes is enhanced by Tfgb_A (12, 52). Apoptosis of osteoblasts requires Bax and is inhibited by Bcl-2. We included this detail as it has been shown that PTH has antiapoptotic effects on osteoblasts *via* Runx2 and upregulation of Bcl-2 (21). As in previous models (32, 33), we assumed that OPG is mainly secreted by Ob_m and that RANKL is mainly secreted by Ob_p. However, model parameters can be changed to model other possibilities, if desired.

Inactive osteocytes are activated by loading (LOAD) or PTH to become activated (Ocy_A). Ocy_I also secretes Sost, which inhibits Wnt signaling. Ocy_I, but not Ocy_A, may undergo apoptosis (14).

Activated osteocytes do not secrete Sost and so indirectly increases Wnt activation (53). Activation of osteocytes is a dynamic process, and so, we also include inactivation of Ocy_A. Osteocytes secrete RANKL in either the active or inactive state (54).

Osteoprotegrin is a decoy receptor and reversibly binds to RANKL to inhibit its activity (8) We include degradation of OPG_RANKL as it has been shown that when OPG binds to RANKL they are internalized by an endocytosis pathway and then degraded (55).

We assume a low basal level of PTH, which leads to very low activation of signaling pathways and so include basal turnover. We also model the effect of the circadian rhythm of PTH by including two model events which simulate a peak of PTH in the late afternoon (5:00 p.m.) and second larger peak of PTH during the night (about 2:00 a.m.).

Parathyroid hormone reversibly binds to Ocy_I, which suppresses the secretion of sclerostin (17) and RANKL by osteocytes and inhibits apoptosis of osteocytes.

Parathyroid hormone binds to Ob_p to inhibit differentiation into Ob_m, and we assume that it increases the rate of RANKL secretion and decreases OPG secretion by precursor cells.

Parathyroid hormone binds to Ob_m, and we assume that Ob_m has a much stronger affinity for PTH than Ob_p or Ocy_I. PTH increases the secretion of RANKL and inhibits the secretion of OPG by Ob_m (18). We assume that PTH has no effect on MCSF secretion by osteoblast cells. PTH activates Wnt and CREB when bound to Ob_m. Activated CREB reversibly binds to Runx2, which leads to synthesis of Bcl2 and inhibition of Bax (21). PTH also increases the degradation rate of Runx2 (21) and TGF-β (56). We assume that Ob_m bound by PTH may form new bone and undergo apoptosis.

TGF-β is secreted by Ob_m in an inactive state (Tgfb_I) and is activated by Ocl_m. Tgfb_A is required for differentiation of Ob_pro to Ob_p, but inhibits differentiation of Ob_p to Ob_m (12). Tgfb_A increases the rate of Ob_m maturation into Ocy_I (12, 52). It also increases the rate of RANKL secretion by Ob_p.

We also include Wnt activation by a PTH-independent mechanism and degradation reactions for all the protein species included in the model.

We assumed that mechanical loading occurs at set times for periods of about 30 min and varied the number of loading events per day to represent different levels of physical activity. In our model, loading represents physical exercise rather than the background loading that occurs throughout the day during everyday life (i.e., movement in general and short periods of walking and climbing stairs, etc.). We made this assumption, as vigorous physical activity is much more likely to be osteogenic (57). We assumed that bone mass is maintained by two bouts of loading per day and set these to occur at about 9:00 a.m. and 4:30 p.m. in the simulations. We also examined the effects of three or four bouts of loading per day to represent the effects of increased physical activity, and just one bout of loading to represent a sedentary lifestyle. LOAD either takes the value 0 or 1. The reaction for osteocyte activation by loading (Ocy_I + LOAD → Ocy_A + LOAD) can only take place when LOAD = 1. We did not include direct effects of loading on osteoblasts or osteoclasts, but assumed that these cells were indirectly affected by loading *via* the effects on osteocytes. However, these could be added in a future development of the model if required.

We assumed that bone mass has an initial value of 2000 units and that each mature osteoblast/osteoclast can degrade/form 1 unit of bone in each degradation/formation reaction, respectively. We assumed that the ratio of active osteoblasts to active osteoclasts during bone remodeling is approximately 10:1 (39). About 10% of the skeleton is remodeled each year; however, it was only feasible to carry out simulations over 100-day periods due to the time required for stochastic simulation. Therefore, we increased the rate of bone turnover so that 10% of bone is remodeled in about 5 months under normal conditions. The half-life of osteoblasts and osteoclasts is about 12 and 8 days, respectively (58, 59). However, we have simplified the remodeling process and have not included spatial aspects and the time delay involved in movement of osteoblasts to the site of remodeling, but instead have assumed that Ob_m and osteoclasts are immediately active, participate in remodeling, and then undergo apoptosis (or in the case of osteoblasts mature to osteocytes). Therefore, it was necessary for the model that levels of Ob_m and osteoclasts have a half-life of about 1 day.

We mainly used deterministic simulation (LSODA algorithm in COPASI) as this is valid for a model in which bone remodeling takes place in a representative elementary volume of the tissue. However, stochastic effects may lead to heterogeneity in cellular responses, and so, we also explored this possibility. It should be noted that we used low molecular numbers in the models so that it was feasible to do stochastic simulation and, therefore, the model output should be viewed as qualitative rather than quantitative data. It would be possible to scale the model for larger numbers if required to give similar model behavior.

**Figure 1:**
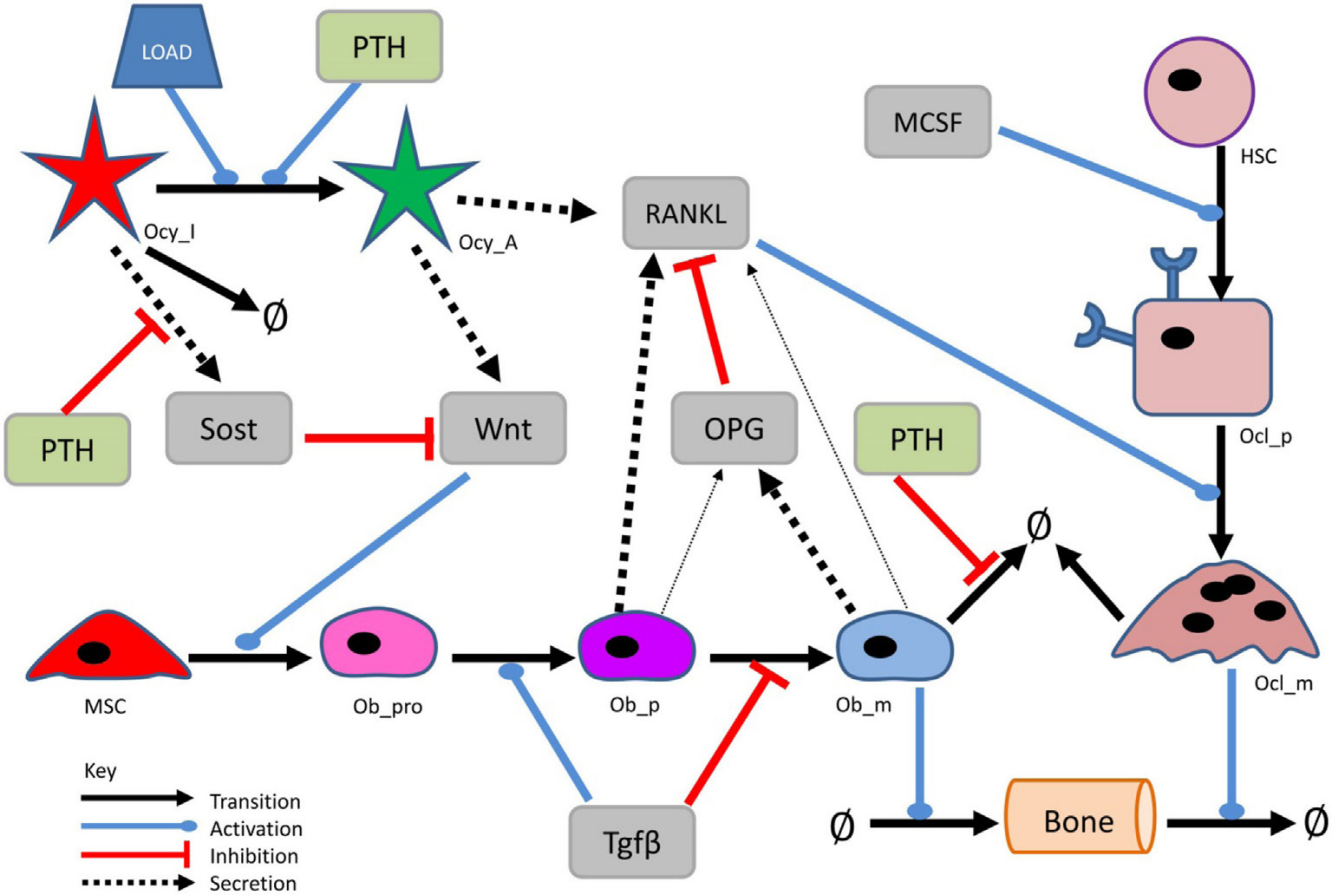
**Simplified network diagram of the model**. Diagram showing the main components and interactions in the model. More detailed diagrams showing all the components of the model are shown in Figures 2–5.

**Figure 2:**
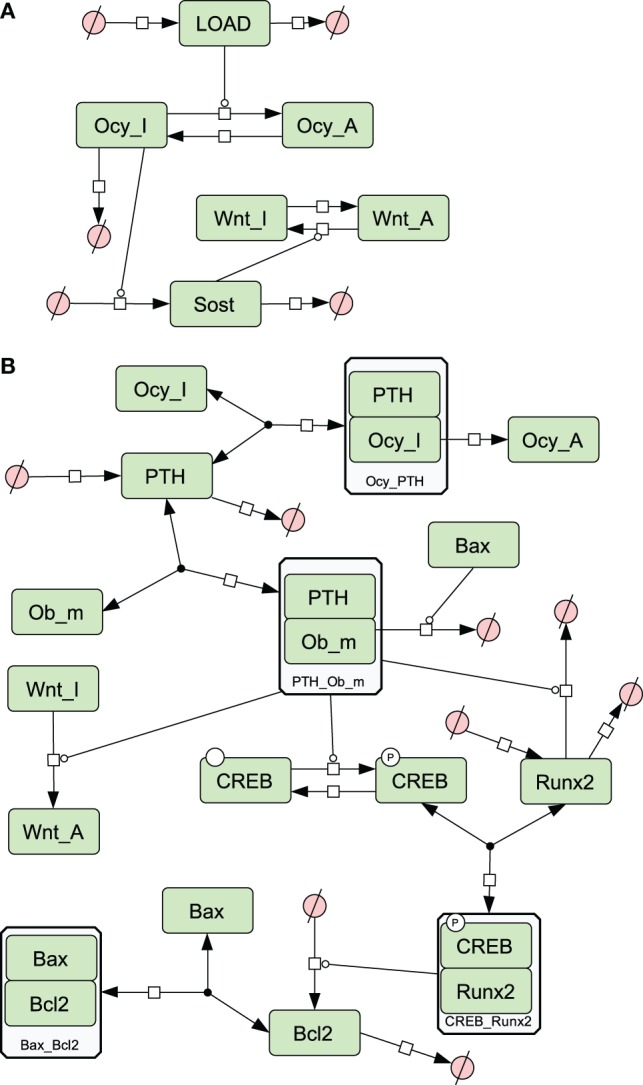
**Network diagrams showing effects of loading and PTH on bone**. **(A)** Activation of osteocytes and Wnt signaling by load; **(B)** effects of PTH on osteocytes and osteoblasts. Figures 2–5 were constructed in CellDesigner using the SBGN. See in Section “Materials and Methods,” modeling assumptions for further details.

**Figure 3:**
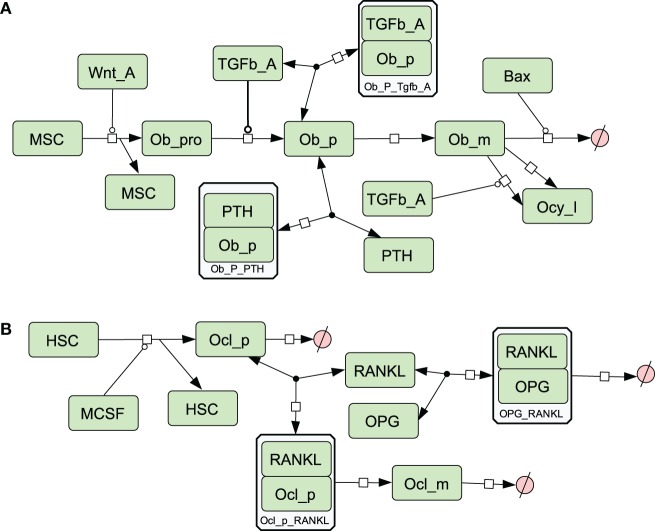
**Network diagram showing species and reactions involved in osteoblast and osteoclast differentiation (A) Osteoblast differentiation; (B) osteoclast differentiation**. See in Section “Materials and Methods,” modeling assumptions for further details.

**Figure 4:**
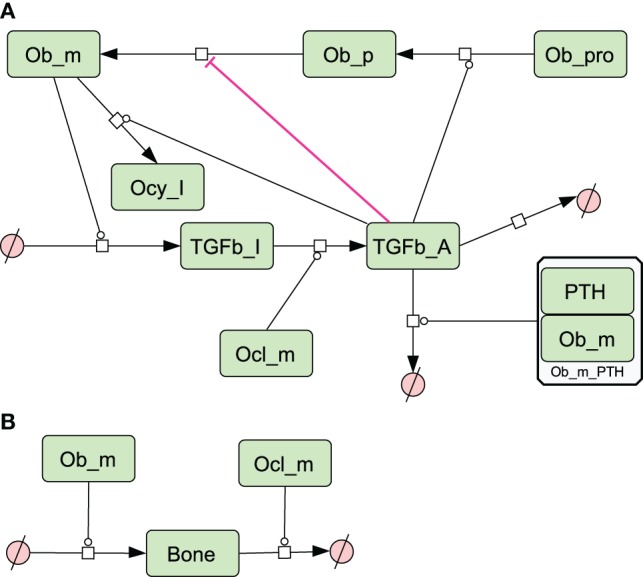
**Network diagram showing species and reactions affected by TGF-β and bone turnover**. **(A)** TGF-β is secreted by osteoblasts, activated by osteoclasts and affects osteoblast differentiation. PTH also enhances degradation of TGF-β; **(B)** bone is formed by osteoblasts and degraded by osteoclasts. See in Section “Materials and Methods,” modeling assumptions for further details.

**Figure 5:**
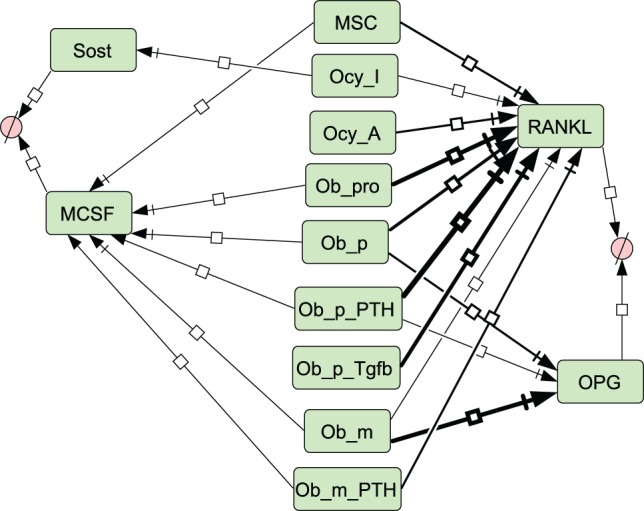
**Network diagram showing secretion of molecules**. Thickness of lines indicates relative rates of secretion by different bone types. See Section “Materials and Methods,” modeling assumptions for further details.

A simplified diagram showing the key components of the model is shown in Figure 1, and graphical network diagrams showing all the model species and reactions are shown in Figures 2–5. In addition, full details of the model species is given in Table 1, and further details of the reactions, parameters, events, sensitivity analysis, and parameter scans are included in the supplementary file (Tables S1–S5 in Supplementary Material). We also show simulation output for each subpart of the model over short timescales, so that the dynamics of the molecular interactions can be easily examined (Figures S1–S5 in Supplementary Material).

**Table 1:** **List of model species**.

Species	Description	Database term	Initial amount
Bone	Bone mass		2000
HSC	Hematopoietic stem cell	CL:1001610	100
MSC	Mesenchymal stem cell	CL:0000134	100
Ob_pro	Osteoblast progenitor	CL:0001040	0
Ob_p	Osteoblast precursor	CL:0007010	0
Ob_m	Mature osteoblast	CL:0001039	0
Ocl_p	Osteoclast precursor	CL:0000778	0
Ocl_m	Mature osteoclast	CL:0000779	0
Ocy_I	Inactive osteocyte	CL:0000137	1800
Ocy_A	Active osteocyte	CL:0000137	0
Ob_p_PTH	Ob precursor bound by PTH		0
Ob_p_Tgfb_A	Ob precursor bound by Tgfβ		0
Ob_m_PTH	Ob_m bound by PTH		0
Ocl_p_RANKL	Ocl_p bound by RANKL		0
Ocy_I_PTH	Ocy_I bound by PTH		0
Bax	Proapoptotic protein	Q07812	100
Bcl2	Antiapoptotic protein	P10415	0
Bax_Bcl2	Bax/Bcl2 complex (inhibits Bax)		0
CREB	Cyclic AMP-responsive element-binding protein 1	P16220	100
CREB_P	Phosphorylated CREB		0
CREB_Runx2	CREB/Runx2 complex		0
MCSF	Macrophage colony-stimulating factor 1	P09603	5
RANKL	RANK ligand	O14788	0
OPG	Osteoprotegerin	O00300	0
OPG_RANKL	RANKL bound to OPG		0
PTH	Parathyroid hormone	P01270	10 or 170
Runx2	Runt-related transcription factor 2	Q13950	10
Tgfb_A	Active transforming growth factor beta-1	P01137	5
Tgfb_I	Inactive Tgfβ	P01137	500
Wnt_A	Activated Wnt	P04628	0
Wnt_I	Inactive Wnt		200
Sost	Sclerostin	Q9BQB4	0
LOAD	Mechanical stimulation		0
X	Dummy species for timing events		0

## Results

### Model Output Shows that Maintenance of Bone Mass Requires Both Regular Loading and a PTH Circadian Rhythm

**Figure 6:**
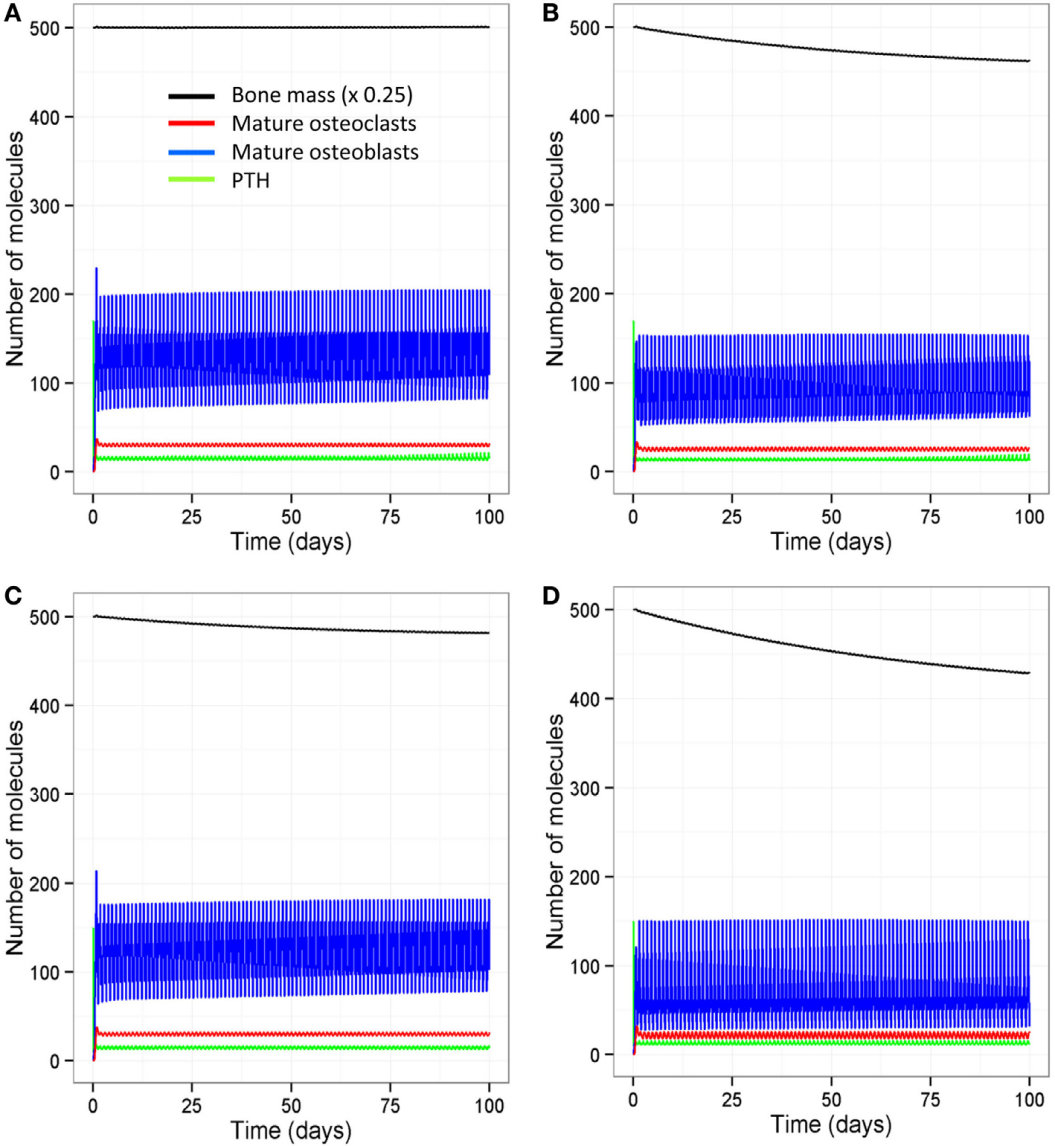
**Effect of loading and an intact PTH circadian rhythm on bone homeostasis**. **(A)** Model output shows that two loading events per day and an intact circadian rhythm of PTH with two peaks per day leads to maintenance of bone mass; **(B)** a decrease in loading (one event per day) with two PTH peaks per day leads to a decline in bone mass with time; **(C)** a decrease in PTH (only one peak per day) with two loading events leads to decline in bone mass with time; and **(D)** a decrease in both loading and PTH (one loading event and one PTH peak per day) leads to a greater decline in bone mass.

**Figure 7:**
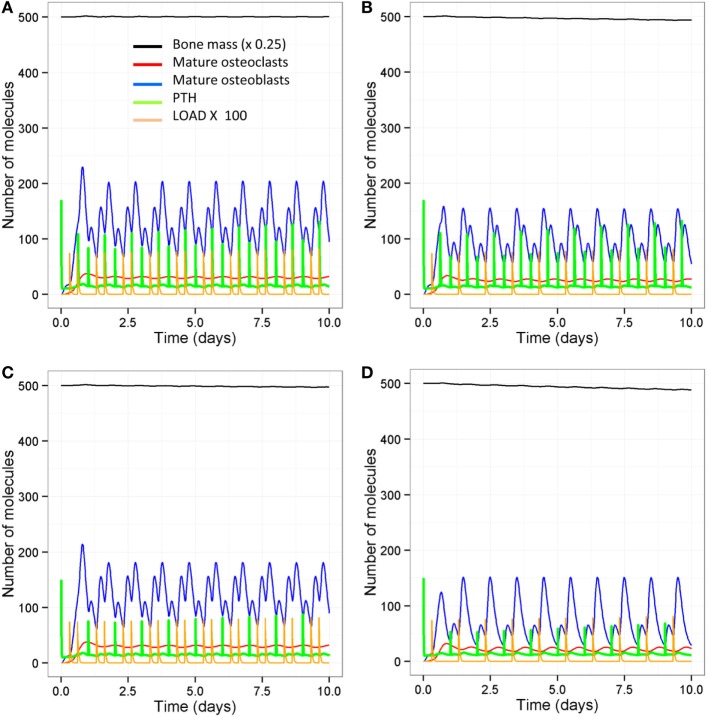
**Simulations over 10-day periods to show effect of loading and PTH events on osteoblast and osteoclast numbers**. **(A)** Model with two loading events and two PTH peaks per day; **(B)** one loading event and two PTH peaks per day; **(C)** two loading events and one PTH peak per day; **(D)** one loading event and one PTH peak per day. Osteoblast levels peak after each loading or PTH event, and so reducing the number of either loading events **(B)** or PTH events **(C)** reduces the frequency and maximum level of the osteoblast peaks. Reducing both loading and PTH **(D)** further reduces the frequency of osteoblast peaks.

The model output shows that bone mass is maintained by regular intermittent loading (two 30-min bouts per day) and a normal PTH circadian rhythm (two peaks per day) (Figure 6A). If loading is reduced to one bout per day with a normal PTH circadian cycle, then bone mass gradually decreases with time (Figure 6B). If PTH only peaks once per day with two bouts of loading per day, bone mass also decreases by a similar amount (Figure 6C). Examination of the model output showed that, immediately after loading and/or increased levels of PTH, there was an overall increase in bone turnover due to an increase in both Ocl_m and osteoblasts. However, there was more bone formation due to a greater increase in osteoblasts and hence, an increase in the osteoblast/osteoclast ratio (Figure 7).

### The Absence of Loading and/or a Complete Disruption of the PTH Circadian Rhythm Results in Severe Bone Loss

**Figure 8:**
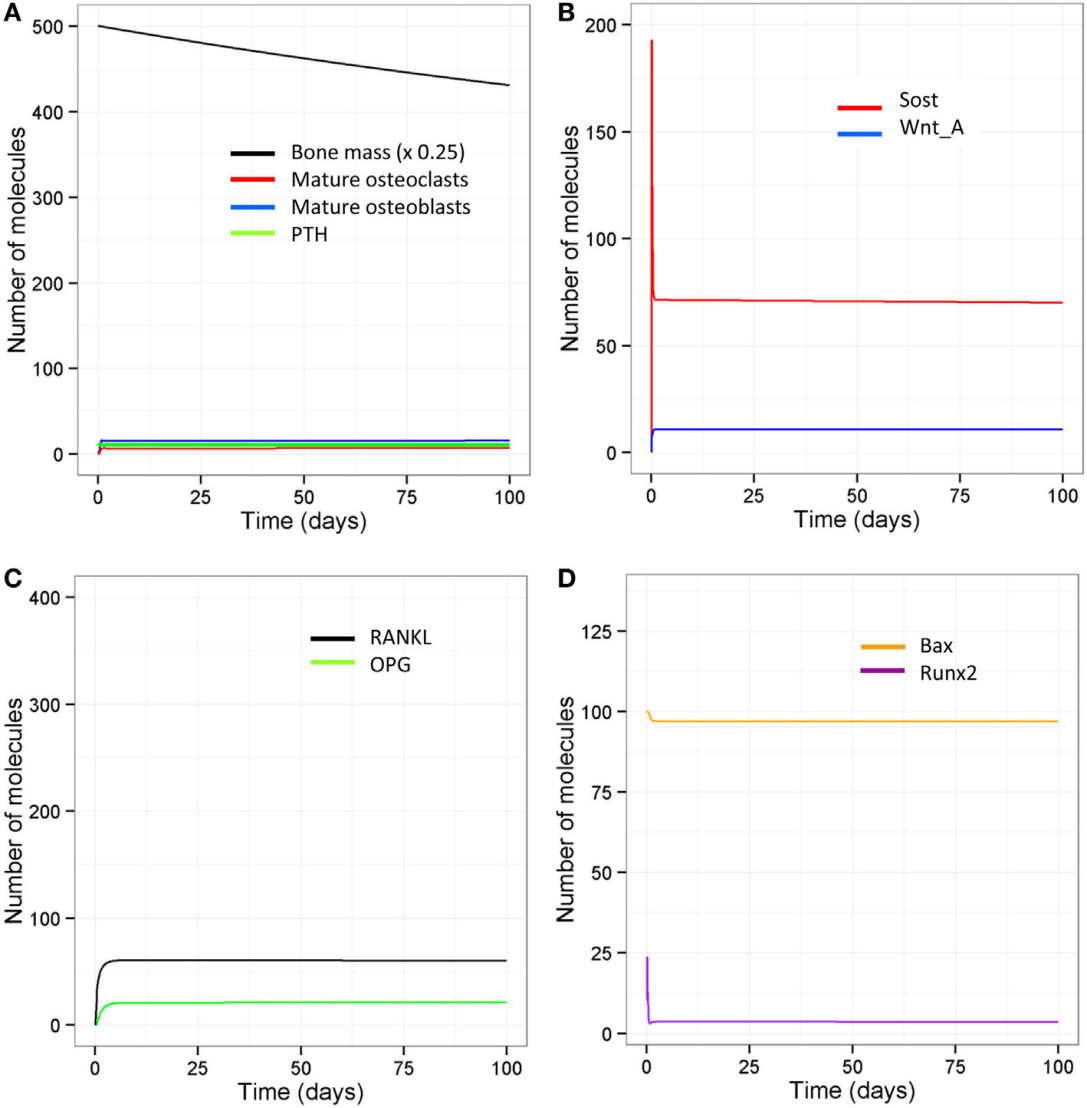
**Model output when there is no loading and no PTH peaks**. **(A)** Bone mass declines steadily with age and there is very little bone turnover as shown by low levels of osteoblasts and osteoclasts; **(B)** Sost levels remain high and so Wnt is mainly inactive; **(C)** RANKL and OPG levels are both lower, but with an overall increase in the RANKL/OPG ratio; **(D)** Bax levels remain high indicating that osteoblast apoptosis is high under these conditions.

With aging, there is likely to be a decline in levels of physical activity and dysregulation of circadian cycles, including PTH, which will have major consequences for bone remodeling. Other possibilities are a complete absence of loading but with an intact PTH cycle, e.g., injury or sickness, leading to prolonged bed rest; or a disrupted PTH cycle but with normal loading, e.g., in disease such as hypoparathyroidism, or an elderly person who is very physically active. The model was simulated under four scenarios: a decline in loading (one event per day) and absence of the early evening PTH peak, no loading with an intact PTH circadian rhythm, normal loading but a totally disrupted PTH cycle (no peaks), and no loading and a totally disrupted PTH cycle. The model output shows that all four scenarios lead to a loss of bone mass over time (Table 2, Figure 6D). A reduction in loading leads to a greater bone loss due to a decrease in the osteoblast/osteoclast ratio. Also, of note, when there is no loading or PTH peaks, there is very little bone remodeling, as seen by the low levels of osteoblasts and osteoclasts (Table 2). This is due to high levels of Sost, which inhibits Wnt signaling, an increase in the RANKL/OPG ratio, and high levels of Bax (Figure 8). This would suggest that, although there is no greater loss in bone mass in the complete absence of physical activity or intermittent PTH peaks, the remaining bone is likely to contain more micro-fractures leading to a greater risk of fracture.

**Table 2:** **Effect of reducing load and/or PTH on bone turnover**.

LOAD (number of events per day)	1	0	2	0
PTH (number of peaks per day)	1	2	0	0
Mature osteoblasts (mean level)	73.8	47.9	102.0	15.3
Mature osteoclasts (mean level)	21.8	14.9	27.1	6.6
Osteoblast/osteoclast ratio	3.39	3.21	3.76	2.32
Bone mass at day 100	1714.1	1722.6	1821.5	1724.3

### Loading and PTH Maintain Bone Mass *via* Increased Wnt Signaling, a Decrease in RANKL/OPG Ratio and a Decrease in Osteoblast Apoptosis

**Figure 9:**
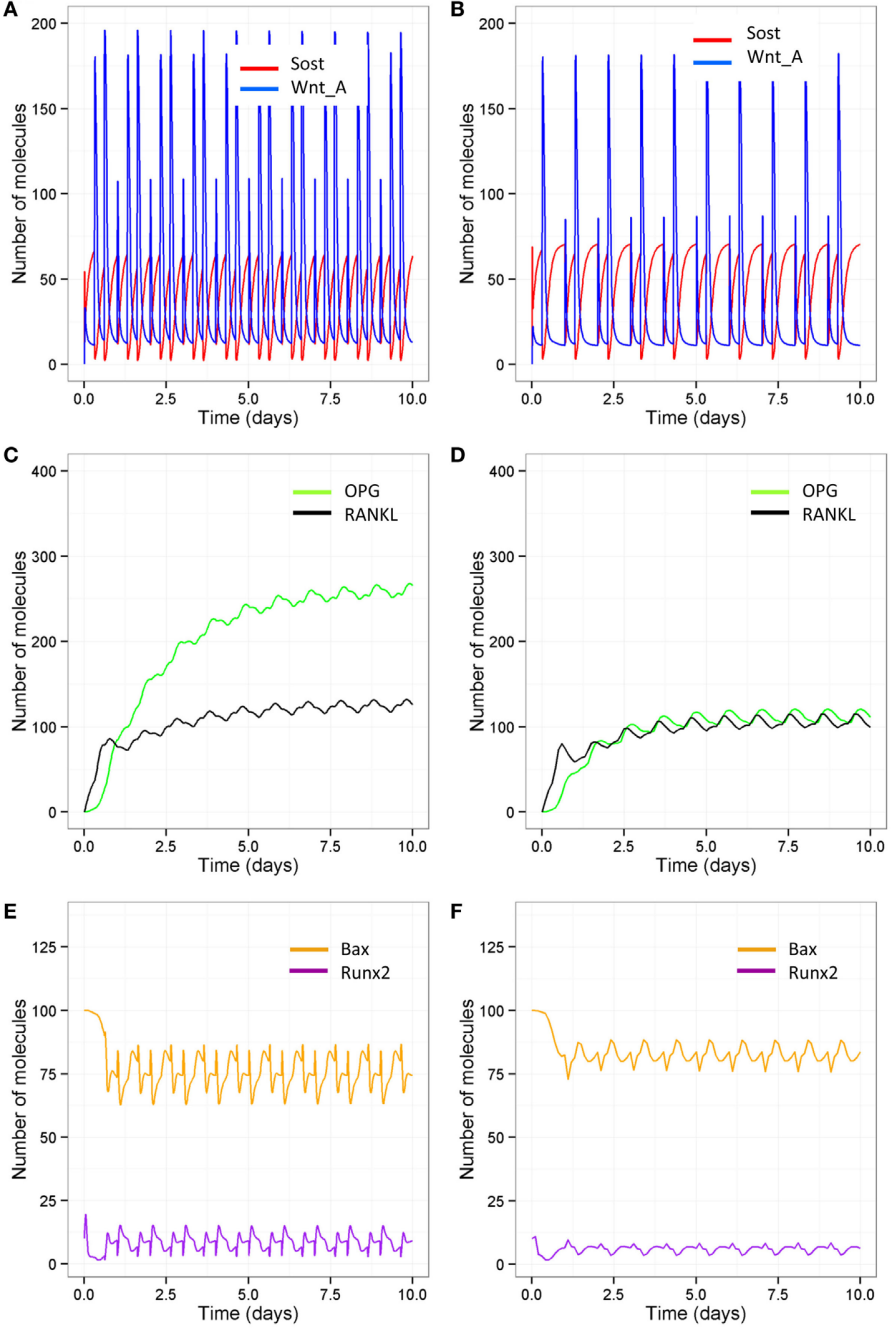
**Model output showing effects of reduced loading and a disruption in the PTH circadian on signaling pathways over 10-day time period**. Comparison of model with two loading events and two PTH peaks per day **(A,C,E)** and model with one loading event and one PTH peak per day **(B,D,F)** show that reduced loading and PTH leads to **(A,B)** higher Sost levels and less activated Wnt; **(C,D)** less OPG and, therefore, a higher RANKL/OPG ratio; and **(E,F)** less Runx2 activity and higher levels of Bax.

**Figure 10:**
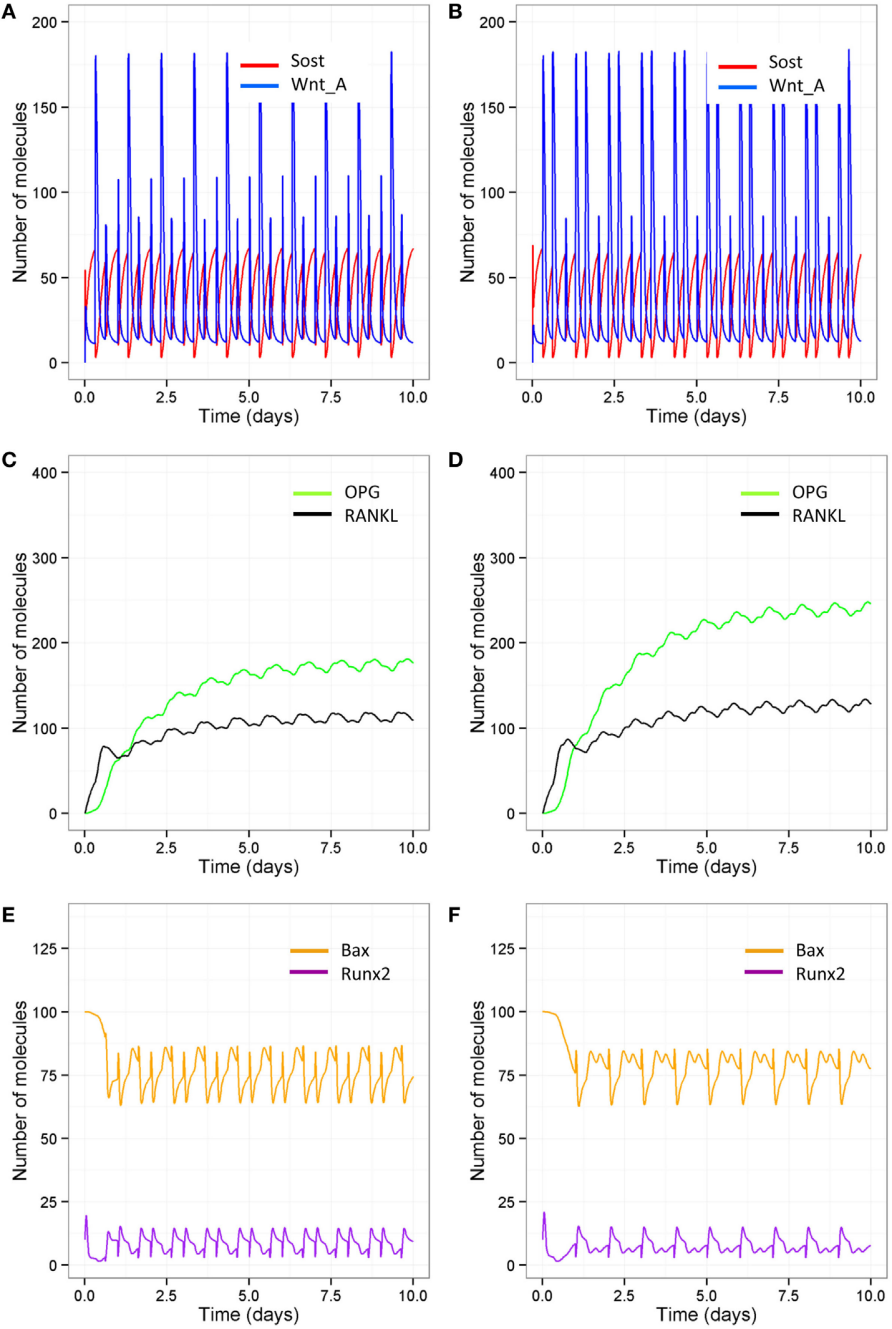
**Model output showing effects of either reduced loading or a disruption in the PTH circadian on signaling pathways over 10-day time period**. Comparison of model with one loading event and two PTH peaks per day **(A,C,E)** and model with two loading events and one PTH peak per day **(B,D,F)** show that reduced loading leads to **(A,B)** less Wnt activation compared to reduced PTH; **(C,D)** less OPG and, therefore, a higher RANKL/OPG ratio; **(E,F)** but reducing PTH leads to less Runx2 activity and higher levels of Bax.

As signaling takes place over short timescale, we also ran simulations over 10-day periods, so that it is easier to distinguish the different patterns in Wnt activation between the different models (Figures 9A,B). After each loading or PTH event, there is an increase in osteocyte activity so that Sost levels decline and Wnt signaling increases (Figure 9A); with only one loading event per day and only one PTH peak per day, Wnt signaling is reduced (Figure 9B). The increase in Wnt signaling accounts for the increase in osteoblast differentiation (Figure 6A). To examine the relative effects of loading and PTH on Wnt signaling, we examined levels of Sost and Wnt when either just loading was reduced or just PTH was reduced. In both cases, there was a similar reduction in Wnt activation (Figures 10A,B). Loading and PTH also led to an increase in both OPG and RANKL, with a decrease in the RANKL/OPG ratio (Figures 9C,D). A reduction in loading only or a reduction in PTH only led to a similar increase in the RANKL/OPG ratio (Figures 10C,D), but the effects were due to different pathways, as there was a synergistic effect when both loading and PTH were reduced (Figure 9D).

In order to examine the effects of loading and PTH on levels of Bax, it was also more informative to look at output from simulations on short timescales as the effects were transient. It can be seen that Bax levels fluctuate, and the minima correspond to the times that PTH levels peak. When there is a normal PTH circadian rhythm, loading does not have much effect on levels of Bax (compare Figure 9E with Figure 10E). However, when there is only one PTH peak per day, additional loading reduces levels of Bax (compare Figure 9F with Figure 10F). Therefore, there is a transient decline in osteoblast apoptosis after each PTH event, which may be exacerbated by loading. Although, loading did not directly affect Bax, the effects were mediated *via* an increase in the number of osteoblasts due to increased Wnt signaling. This led to more PTH binding to osteoblasts to enhance Runx2 signaling and synthesis of Bcl2, which bound to Bax to inhibit its activity.

### Simulation of Interventions Suggests Treatments to Prevent Bone Loss in Aging

To simulate interventions, we used the model with one loading event and one PTH peak per day, as this model mimics the situation in aging with reduced physical activity and a disrupted PTH circadian rhythm with a significant loss of bone mass over a 100-day period.

### Simulation of RANKL Inhibition

**Figure 11:**
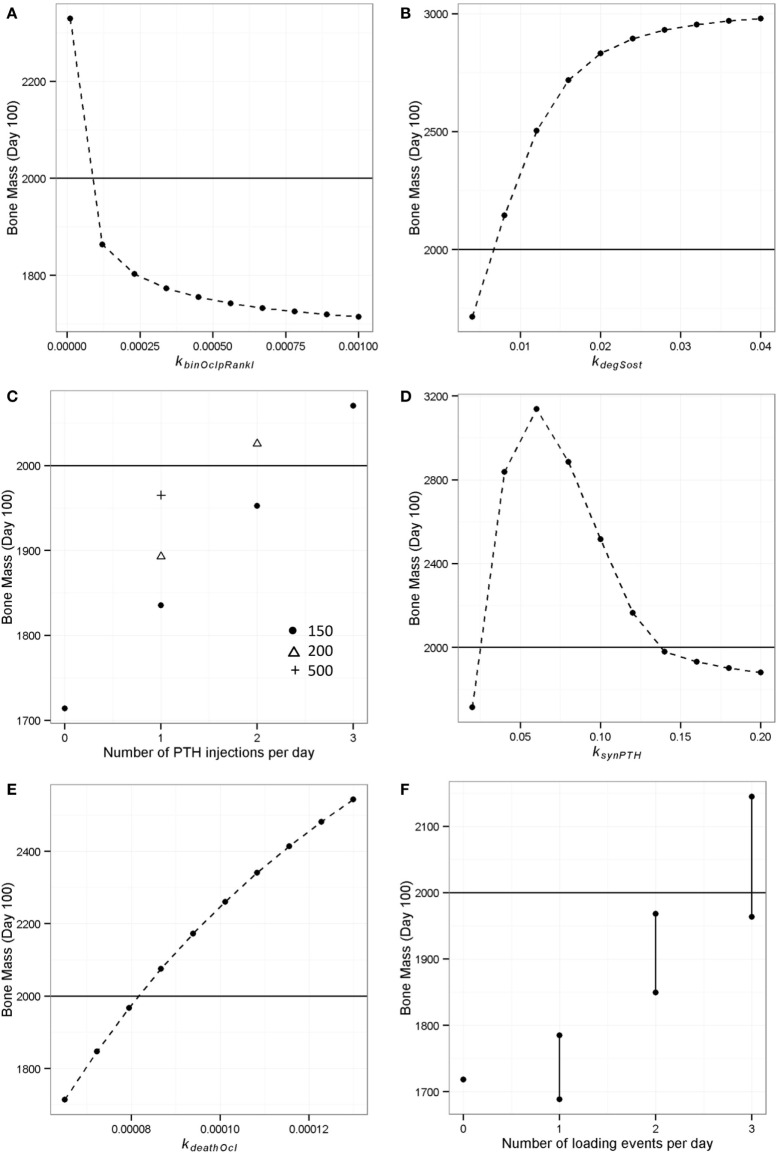
**Model output showing effect of simulated interventions on bone remodeling**. The deterministic model of “simulated aging” (one loading event and one PTH peak per day) was used to simulate interventions and the bone mass on day 100 was recorded to see which interventions could restore the loss of bone mass (i.e., a bone mass of 1714) to “normal” levels (i.e., 2000, indicated by horizontal line in each graph). **(A)** RANKL inhibition was simulated by decreasing the binding rate of RANKL to osteoclasts from 0.001 to 0.0001; **(B)** Sost inhibition was simulated by increasing the rate of Sost degradation from 0.004 to 0.04; **(C)** PTH injections were simulated by including additional events in the model, and different doses were simulated by varying the value of the PTH peak at the event; **(D)** continuous PTH administration was simulated by increasing the rate of PTH synthesis from 0.02 to 0.2; **(E)** bisphosphonate treatment was simulated by increasing the rate of osteoclast apoptosis; **(F)** increased physical activity was simulated by including additional events for loading, the effect depended on the timing of the loading events and vertical lines show the range of values obtained.

The RANKL inhibitor, denosumab has recently been introduced as a new treatment for osteoporosis and has been shown to increase bone mass in postmenopausal women (60). In order to simulate the effects of RANKL inhibition, we decreased the value of the parameter that controls the rate of RANKL binding to its receptor on osteoclasts (*k_binOclpRankl_*) from 0.001 to 0.0001 in steps of 0.0001. This led to a gradual increase in bone mass, and at the lowest value, bone mass increased to just above its normal value (Figure 11A).

### Simulation of Sost Inhibition

Sost inhibitors are currently being tested as potential treatments for osteoporosis and show promise as an anabolic agent (61). To simulate the inhibition of Sost, we increased the value of the degradation rate of Sost (*k_degSost_*) from its default value of 0.004 to 0.04, in steps of 0.004. This led to a rapid increase in bone mass as *k_degSost_* initially increased and then flattened off, suggesting a maximal effect for this intervention (Figure 11B).

### Simulation of PTH Injections

The anabolic effect of daily PTH injections has been well documented, but the effects of PTH on bone metabolism are complex and the underlying mechanisms are not well understood (62). We simulated the effect of giving one, two, or three PTH injections per day. In the case of one injection, this was simulated to occur in the late afternoon (at the time that PTH would normally peak). One injection was insufficient to restore bone mass even if we increased the amount of the PTH peak (Figure 11C). However, an additional injection was able to restore bone mass if the injected amount of PTH was set to 200, and with three injections, a low dose was sufficient (Figure 11C).

### Simulation of Continuous PTH

It has been shown that administration of PTH by pump delivery can increase bone mass in children suffering from severe hypoparathyroidism (63), but many studies show that continuous administration of PTH can lead to bone loss, e.g., Ref. (26). Therefore, we ran simulations to see if our model could capture this behavior by increasing the value of *k_synPTH_*, which had the effect of increasing the basal levels of PTH. As observed experimentally, our model predicted that there is an optimal level of PTH with either low levels or continuous high levels being detrimental (Figure 11D).

### Simulation of Effects of Bisphosphonates

Bisphosphonates inhibit the degradation of bone by increasing the rate of osteoclast apoptosis or inhibiting osteoclast differentiation (64) and are the most used drug for the treatment of osteoporosis in the UK. We simulated the effect of administering bisphosphonates at increasing doses by increasing the rate of osteoclast apoptosis (*k_deathOcl_*) from 6.5e−5 to 1.3e−4 (Figure 11E). This led to an increase in bone mass with no maximal effect being apparent.

### Simulation of Effect of Additional Loading

**Figure 12:**
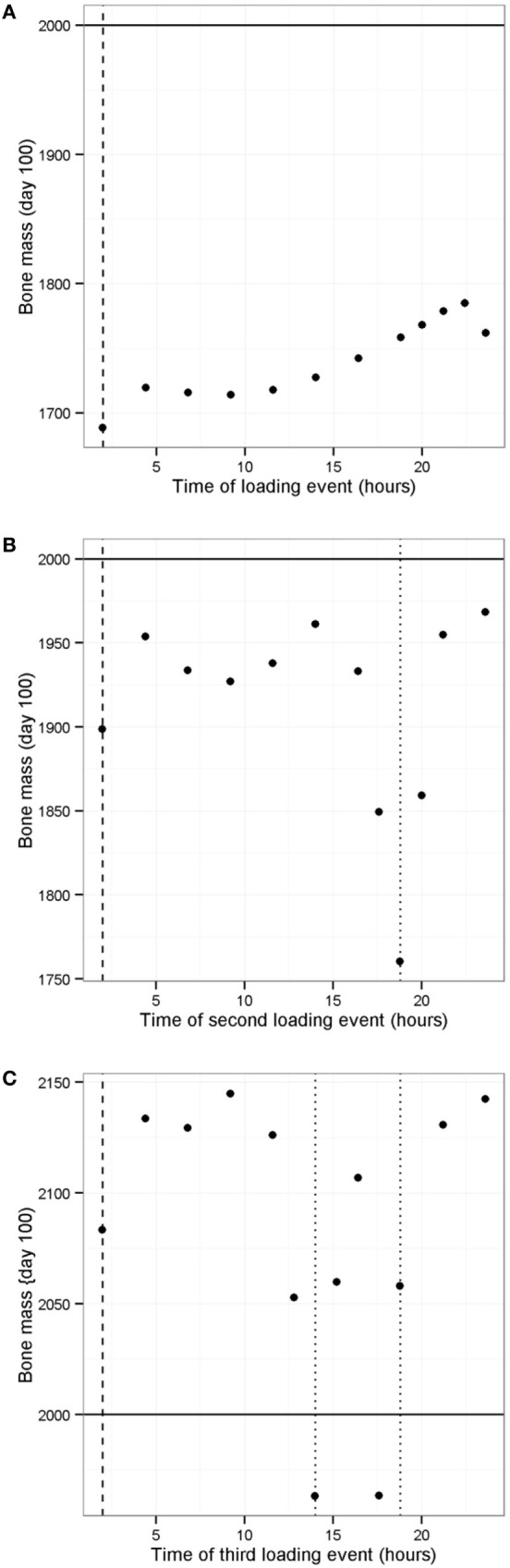
**Effect of timing of loading events on bone mass**. **(A)** Model with one PTH peak and one loading event per day shows that best time for loading is in the late evening; **(B)** model with one PTH peak per day and one loading event set at 1900 hours, shows that best time for second loading event is at 1400 hours (excluding peak at about 0100 hours); **(C)** model with one PTH peak and two loading events per day (at 1400 and 1900 hours) shows that a third loading event leads to an increase bone mass if it is not close to another loading event. Vertical dashed line shows time of PTH peak, vertical dotted line shows time of loading event(s), solid horizontal line indicates normal bone mass.

It is known that physical activity is important for maintaining bone mass. We used the model to investigate the effect of different loading regimes with zero, one, two, or three loads per day using the model with only one PTH peak per day (at 0200 hours). With only one PTH peak per day, it was necessary to have three loading events per day (Figure 11F). Interestingly, the timing of the additional loads was found to be important (as shown by the range of values in Figure 11F), and the model showed that the best time for a single load was 2100 hours (Figure 12A). Since physical activity close to bedtime may have undesirable effects, we chose 1848 hours for the first load (i.e., the time that gave maximum benefit if night time loading is excluded), when investigating the best time for the second load (Figure 12B). An additional load was least effective if administered close to 1900 or 0200 hours, when loading and PTH peaks were already present (Figure 11F), and the model predicted that the best time for the second loading event was early afternoon or very late evening (Figure 12B). The time of the second load was then fixed at 1400 hours, and the time for the third load was varied. Provided the third load was not close to another loading event, bone mass increased above basal level over a 100-day time period, with maximum benefit occurring if the loading events were about 4–5 h apart (Figure 12C).

### Sensitivity Analysis Shows that Bone Remodeling Is Mainly Affected by Components of the Wnt and PTH Pathways

Sensitivity analysis was carried out in COPASI on the deterministic version of the model with two loading and two peaks of PTH per day, in which bone homeostasis is maintained. The results are shown in File S2 in Supplementary Material. The parameters which affected the final level of bone mass after 100-day simulation are highlighted (green for an increase and red for a decrease in bone mass) and summarized in Table S4 in Supplementary Material of File S1 in Supplementary Material. The sensitive parameters include those involved in osteoblast differentiation (*k_diffMSC_* and *k_diffObp_*), osteoclast differentiation (e.g., *k_diffHSC_*, *k_degMCSF_*, and *k_secRANKLbyOcy_*), the Wnt pathway (e.g., *k_actWnt_*, *k_degSost_*, and *k_secSost_*), the PTH pathway especially the effects of PTH on osteoblast apoptosis (e.g., *k_binCrebRunx2_*, *k_synBcl2_*, and *k_deathOb_*), and those that affected the RANKL/OPG ratio (e.g., *k_secOPGbyObm_*, *k_inhibRANKL_*, and *k_secRANKLbyOcy_*). These parameters were then varied in the range of 0.5–2 times their default values, and the model simulated to examine the effect on all the model components. A summary showing the effects of changing the sensitive parameters on bone mass, Ob_m, Ocl_m, and Ocy_A is shown in Table S5 in Supplementary Material.

### Stochastic Model Indicates that Variability in Bax Levels May Lead to Heterogeneity in Cellular Responses

**Figure 13:**
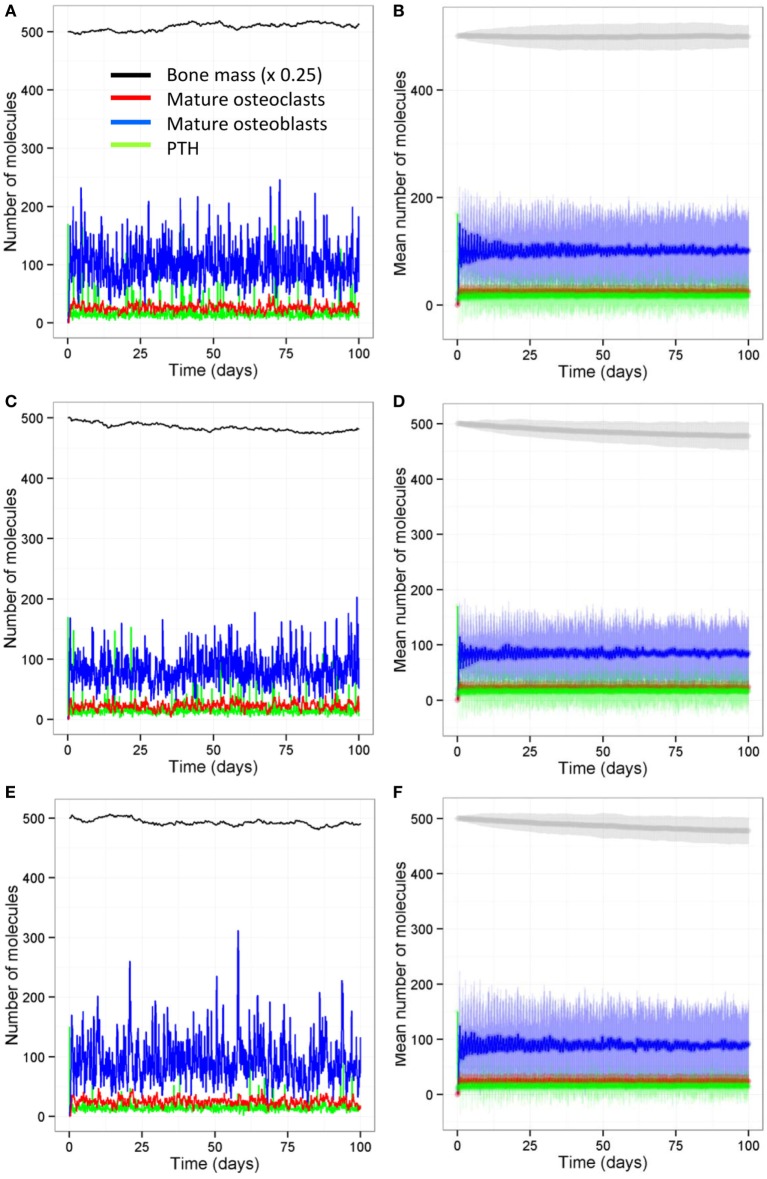

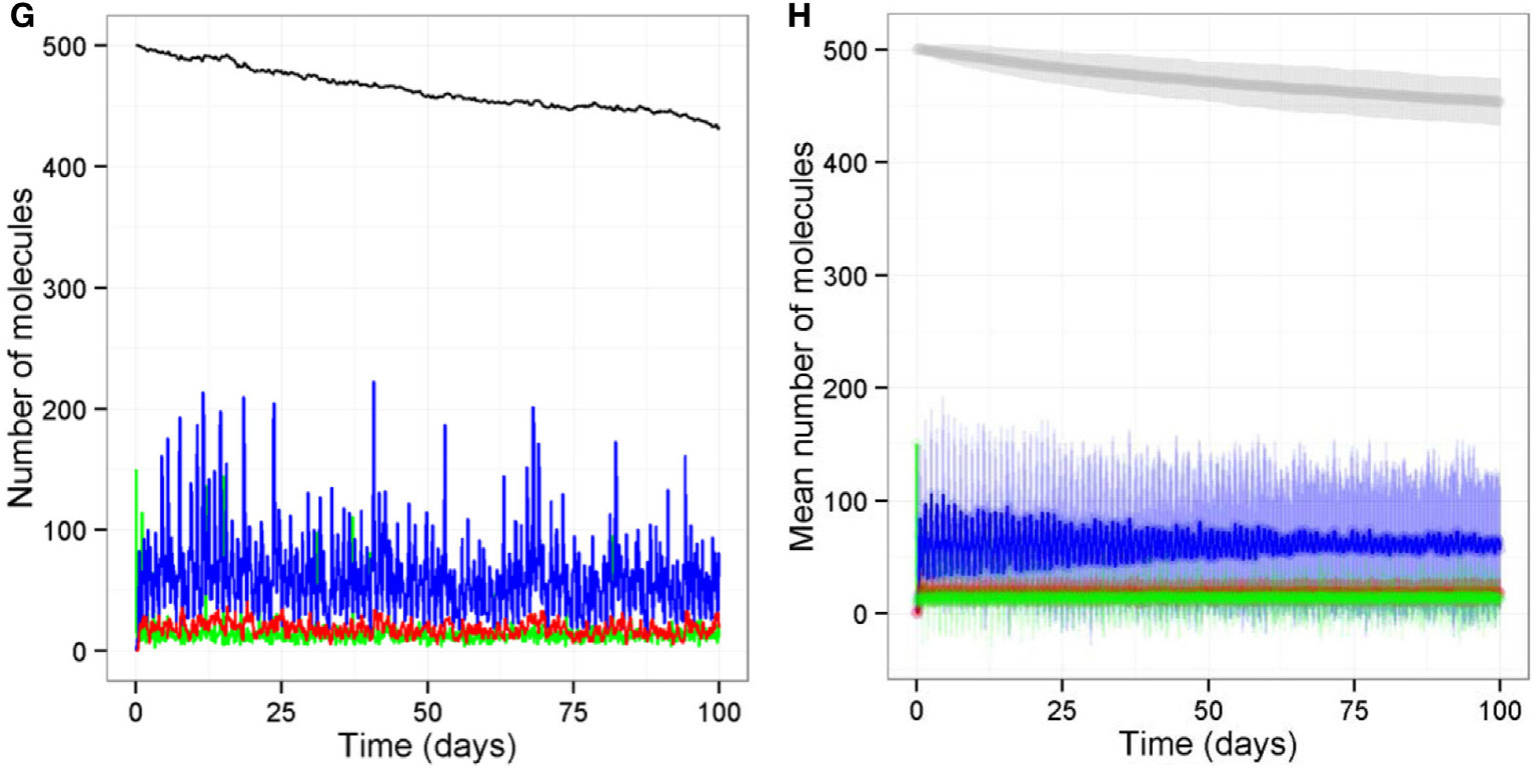
**Stochastic model shows similar behavior to the deterministic models**. **(A,B)** Model with two loading events and two PTH peaks per day; **(C,D)** model with one loading event and two PTH peaks per day; **(E,F)** model with two loading events and one PTH peak per day; **(G,H)** model with one loading event and one PTH peak per day. Output from the stochastic model shows similar behavior to the deterministic model (Figure 6). **(A,C,E,F)** show output from one individual stochastic simulation; **(B,D,F,H)** show the mean levels (dark curves) and ±1.96 SDs from the mean (light shaded region) from 100 individual simulations.

**Figure 14:**
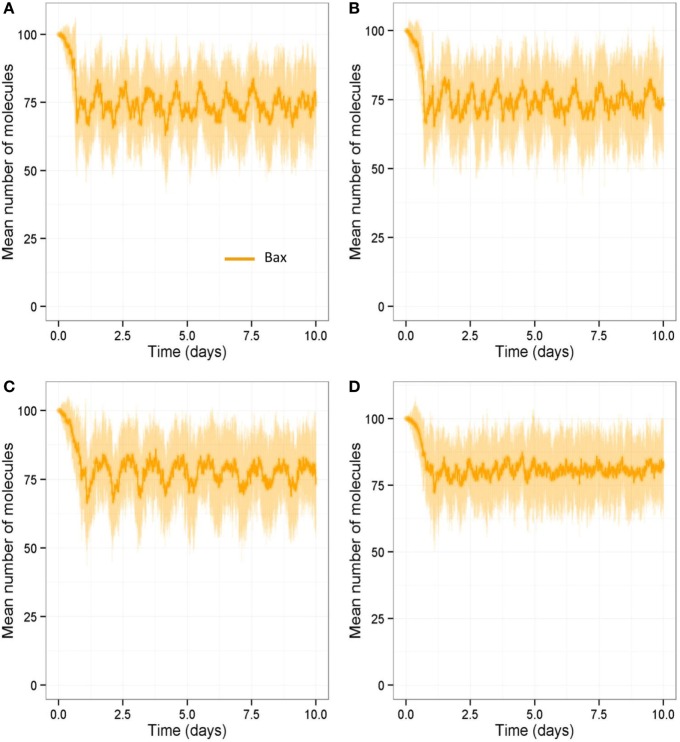
**Stochastic simulations showing effect of loading and PTH on Bax over 10-day period**. Model output over 10-day period shows that Bax levels are more variable and are low for longer periods of time when PTH peaks are present. **(A)** With two loads and two PTH events per day, intermittent Runx2 activity leads a decline in Bax levels; **(B)** a reduction in loading (one load per day) does not show any obvious difference in Bax levels; **(C)** a reduction in PTH (one peak per day) leads to higher Bax levels with fewer dips in Bax levels to below 50 (half its basal level); **(D)** a reduction in both loading (one load per day) and PTH (one peak per day) leads to much higher Bax levels, and it rarely dips below 50. **(A–D)** show output from one individual stochastic simulation.

We also ran all the models with a stochastic algorithm (Gillespie direct method) to investigate whether stochastic effects are important in bone remodeling. Overall the results were very similar to the deterministic output (compare Figure 13 to Figure 6). However, it can be seen from the SD of 100 simulations that there is variability in the amount of bone mass at day 100. We also noted that intermittent PTH peaks affected the variability of Bax levels with Bax being more variable and lower for longer periods of time, when PTH peaks are present (Figure 14). So when PTH peaks, Bax levels decline, and so, there is a delay in osteoblast apoptosis. There is variability in both the minimum level of Bax and the length of time that it is below a threshold required for apoptosis between simulations, and so there will be variability in the lifespan of mature osteoblasts.

## Discussion

We have used computational modeling to examine some of the key processes involved in bone remodeling. We included the differentiation of osteoblasts and osteoclasts and the major signaling pathways involved in these processes. We also included osteocytes as they respond to mechanical stimuli and secrete signaling molecules, which are important in bone remodeling. We assumed that PTH is normally present in the extracellular environment at low basal levels, which are below the threshold required for cell signaling, but that it is has a circadian rhythm, whereby it peaks twice a day. The main purpose of the model was to examine the effects of loading and the PTH circadian rhythm on bone homeostasis and to provide a basic model, which we can extend in the future to examine the effects of loading and PTH in more detail in combination with other signaling pathways, so that it can be used to examine possible new therapeutic interventions or combinations of therapies. In addition, we tested whether our model would predict opposing effects of continuous or intermittent PTH treatments on bone remodeling as has been previously shown experimentally and in previous mathematical models.

The model parameters for bone turnover were adjusted so that bone mass is maintained over time with two loading events and an intact PTH circadian rhythm with two peaks per day, although there were fluctuations in bone mass over the time course due to bone remodeling taking place. Either a decline in loading (one event per day) or disruption to the PTH cycle (just one peak per day) led to bone mass gradually declining with time. Examination of the model output showed that this was mainly due to higher levels of sclerostin and inhibition of Wnt signaling, as has been shown experimentally (65). Loading and PTH also increased the levels of OPG so that the RANKL/OPG ratio decreased leading to an increase in the ratio of Ob_m to Ocl_m, and hence, more bone formation took place after each loading or PTH event. As expected, after each PTH event, there was an increase in Runx2 activity leading to temporary decline in Bax and, therefore, less osteoblast apoptosis for short periods. This was also the case after each loading event due to the increase levels of Ob_m and more PTH binding to osteoblasts to initiate Runx2 signaling. Loading also led to slightly higher levels of PTH due to a decrease in PTH degradation, since degradation only takes place once PTH has been internalized. We modeled this by assuming that PTH is released from the osteoblast and then degraded, but a future modification of the model could include PTH receptors so that receptor-bound PTH is internalized.

We carried out a sensitivity analysis to investigate which model parameters had positive or negative effects on bone mass, as this would not only confirm that the expected behavior based on prior knowledge of bone remodeling but would also suggest potential targets for interventions. The most sensitive parameters were those that affected the lifespan of osteoblasts or osteoclasts and the differentiation process for both bone cells. Parameters involving all the signaling pathways included in the model were also important with no particular pathway being dominant. Therefore, our model suggests that interventions involving multiple targets may be more effective than a single target. This would require further investigation, and computational models will be invaluable in the future to help suggest which combination of targets would be appropriate for experimental testing.

Our model (in which there was only one loading event and PTH peak per day) reproduced the effects of continuous versus intermittent PTH, with the former intervention leading to bone loss if the concentration of PTH is high, and the latter able to maintain bone mass if administered twice daily. Continuous PTH at intermediate levels was able to maintain bone mass. This was due to an increase in Wnt signaling. However, high levels of continuous PTH led to loss of bone mass, as continuous PTH both reduced Runx2 activity leading to an increase in osteoblast apoptosis and increasedthe RANKL/OPG ratio leading to an increase in osteoclast differentiation. On the other hand, the model showed that intermittent PTH maintained bone mass due to increased Wnt signaling and the activation of CREB and Runx2, which led to temporary decline in osteoblast apoptosis after each simulated “injection.” The temporary effects of PTH on osteoblast apoptosis was due to the fact that PTH also enhanced the degradation of Runx2 to provide a negative feedback loop to terminate signaling. The complex effects of PTH means that using treatments, such as teriparatide, require caution, as is already known (66). Our model suggests that whether or not the patient has an impaired PTH circadian rhythm should also be investigated in order to avoid increasing bone loss or at the other extreme to increase the risk of bone tumors. For example, it has been shown that the timing of teriparatide injections is important in postmenopausal osteoporosis with regards to the natural circadian rhythm of bone resorption and effectiveness of treatment (67, 68).

In addition to PTH treatments, we modeled the effects of other interventions, which are currently in use or being tested as a potential treatment for osteoporosis. Our model was able to reproduce the effects of RANKL inhibitors, Sost inhibitors, and bisphosphonates. We also examined the effects of increased physical activity by varying the number of loading events per day. Interestingly, our model predicted that the timing of physical activity may be important. This is mainly due to the natural circadian rhythm of PTH and also the fact that both PTH and loading lead to transitory signaling due to negative feedback loops within signaling pathways. Therefore, short, regular intervals of activity may be more beneficial than one prolonged exercise period per day. In addition, the benefits of exercise may be more pronounced if taken at times when PTH levels are normally low. It would be interesting to test these predictions in a clinical setting or an animal model of osteoporosis.

We modeled the effect of loading by simply assuming that load activates osteocytes, which in turn activates Wnt signaling and osteoblast differentiation. It is known that purinergic signaling also plays an important role in bone remodeling and that both osteoblasts and osteoclasts express P2 receptors (23). It is also known that osteocytes, osteoblasts, and osteoclasts secrete ATP in response to loading (69–71). Therefore, a proposed extension to this model is to include the direct effects of loading on all bone cells. Interestingly, the model predicted that timing of the loading events was important for maintenance of bone mass and suggested that spacing the loads throughout the day and at times that are not close to the natural peaks of the PTH circadian rhythm is more beneficial. It would be interesting to test this experimentally by having groups of subjects undergoing different amounts of physical activity using different time schedules. The interaction between physical activity and the PTH circadian rhythm also warrants further study. It also raises the question of whether shift workers and persons who make frequent long haul flights have an accelerated loss of bone mass with age and so are more at risk of osteoporosis. Lastly, the model suggests that a combination of increased physical activity and daily low dose PTH injections may be beneficial.

We modeled the process of bone remodeling over fairly short timescales (100 days) as in previous mathematical models. However, the models could be modified so that they could be run over longer timescales in order to examine changes that take place during aging. For example, it has been shown that levels of PTH have a circadian rhythm, which changes with age (27) and, therefore, it would be interesting to include this aspect of PTH regulation. Other molecular pathways are also known to change with age, such as TGF-β signaling (72) and the OPG/RANKL pathway (73). It is known that stem cells change with age, and so, the model could be used to examine the effects of damage to stem cells on the differentiation processes of osteoblasts and osteoclasts (74, 75). Lastly, the model could be modified to include the effects of different loading regimes, e.g., repetitive and/or damaging by adding reactions to represent damage to bone cells and including different types of loading. Cellular responses to mechanical signals also change with age (76), which may be due to dysregulation of signaling pathways with age. In conclusion, our computer model demonstrates the utility of a computational tool to investigate how age-related changes affect bone remodeling to complement experimental approaches. We have shown how modeling can be used to test possible interventions or combinations of therapies that could prevent or slow down the loss of bone mass with age, and to suggest future studies. In particular, our model suggests that the relationship between mechanical stimulation and the PTH circadian with implications for the timing of interventions requires further investigation.

## Author Contributions

CP constructed the models, ran the simulations, analyzed the results, drafted the manuscript, and approved the final version. AG contributed to the design of the models, interpretation of results, revised the manuscript for important intellectual content, and approved the final version. Both authors agree to be accountable for all aspects of the work in ensuring that questions related to the accuracy or integrity of any part of the work are appropriately investigated and resolved.

## Conflict of Interest Statement

The authors declare that the research was conducted in the absence of any commercial or financial relationships that could be construed as a potential conflict of interest.
