# Supplementary material for: Simulated Interventions to Ameliorate Age-Related Bone Loss Indicate the Importance of Timing
Source: Front Endocrinol (Lausanne). 2016 Jun 13;7:61. doi: 10.3389/fendo.2016.00061 (PMC4904033; doi:10.3389/fendo.2016.00061)
Supplement: Supplementary file 1 [file Presentation_1.PDF]

Supplementary Material

Simulated interventions to ameliorate age-related bone loss indicate the importance of timing

Carole J Proctor[a,b,*], Alison Gartland[a,c]

[a]MRC/Arthritis Research UK Centre for Musculoskeletal Ageing (CIMA), [b]Musculoskeletal Research Group, Institute of Cellular Medicine, Medical School, Newcastle University, Newcastle upon Tyne NE2 4HH, United Kingdom, [c]Department of Oncology and Metabolism, University of Sheffield Medical School, Beech Hill Road, Sheffield, S10 2RX.

*** Correspondence:** Carole J Proctor: carole.proctor@ncl.ac.uk

1. Supplementary Figures
2. Supplementary Tables

Supplementary Figure 1 Model output of sub-model "Loading" This figure shows the output for the model species that are immediately affected by LOAD (see Figure 1 of main manuscript). After each loading event, osteocytes are activated within 1 hour, leading to downregulation of Sost and activation of Wnt. There is then a slower return to basal levels. This agrees with experimental data (Lara-Castillo et al., 2015). LOAD is scaled by 100 for visualisation. Simulations were performed over 2 day period to give a clearer picture of the dynamics.

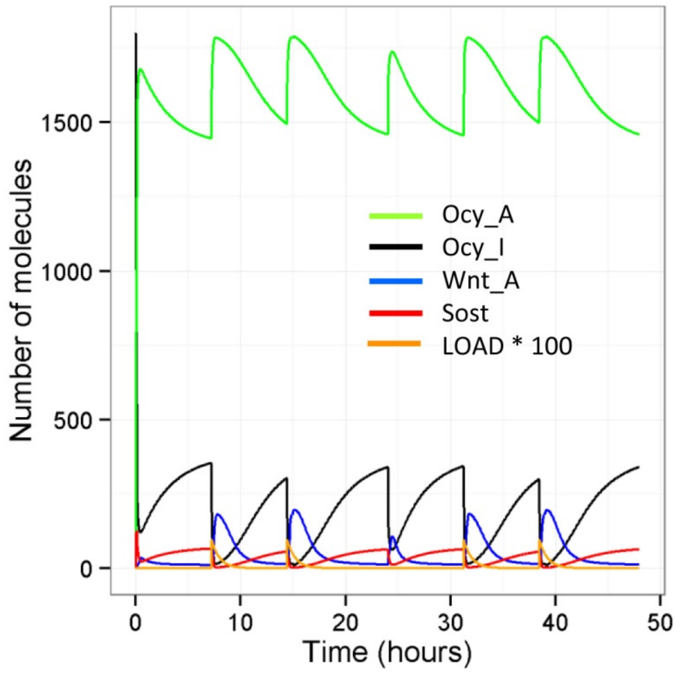

Supplementary Figure 2 Model output of sub-model "PTH". After each PTH event, there is a rapid increase in CREB phosphorylation, leading to an increase in Runx2 and inhibition of Bax. There is a slow return to basal levels, so when peaks of PTH are close together, the second peak of Runx2 is slightly higher, leading to lower levels of Bax – this corresponds to the night-time peak of PTH. Simulations were performed over 2 day period to give a clearer picture of the dynamics. Note actual time of simulations starts at 2 am with a peak in PTH.

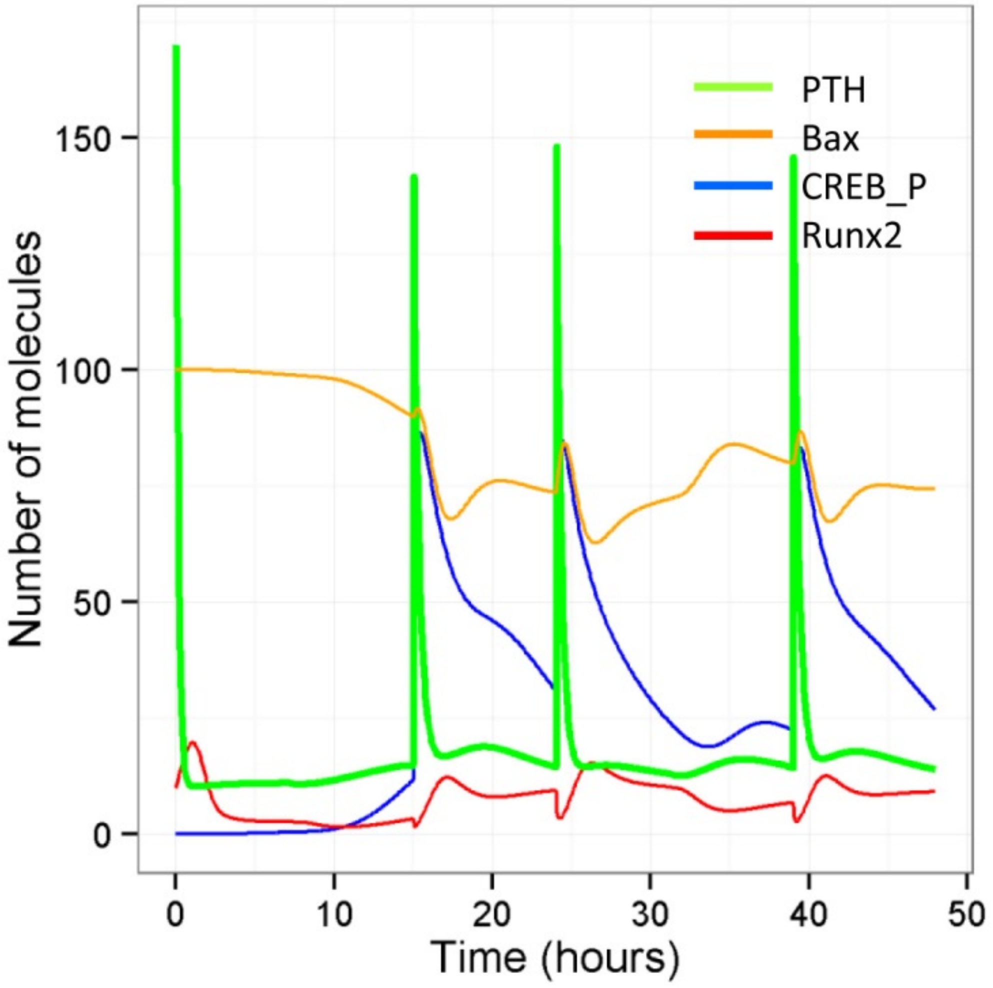

Supplementary Figure 3 Model output of sub-model "Osteoblast differentiation" The loading event at about 7 hours (which corresponds to 9am) results in a rapid activation of Wnt leading to MSCs differentiating into osteoblast progenitors (Ob_pro). This is followed by differentiation into osteoblast precursors (Ob_p) and finally mature osteoblasts (Ob_m). The attenuation of the Ob_pro response after 24 hours is due to the increase in Tgfb_A (which increases over the first 24 hour period and then oscillates – see Supp Fig 5) and therefore an increase in the rate at which Ob_pro differentiates into Ob_p. The lack of a regular pattern is due to the irregular spacing between the events for load and PTH. Simulations were performed over 2 day period to give a clearer picture of the dynamics. Note actual time of simulations starts at 2 am with a peak in PTH.

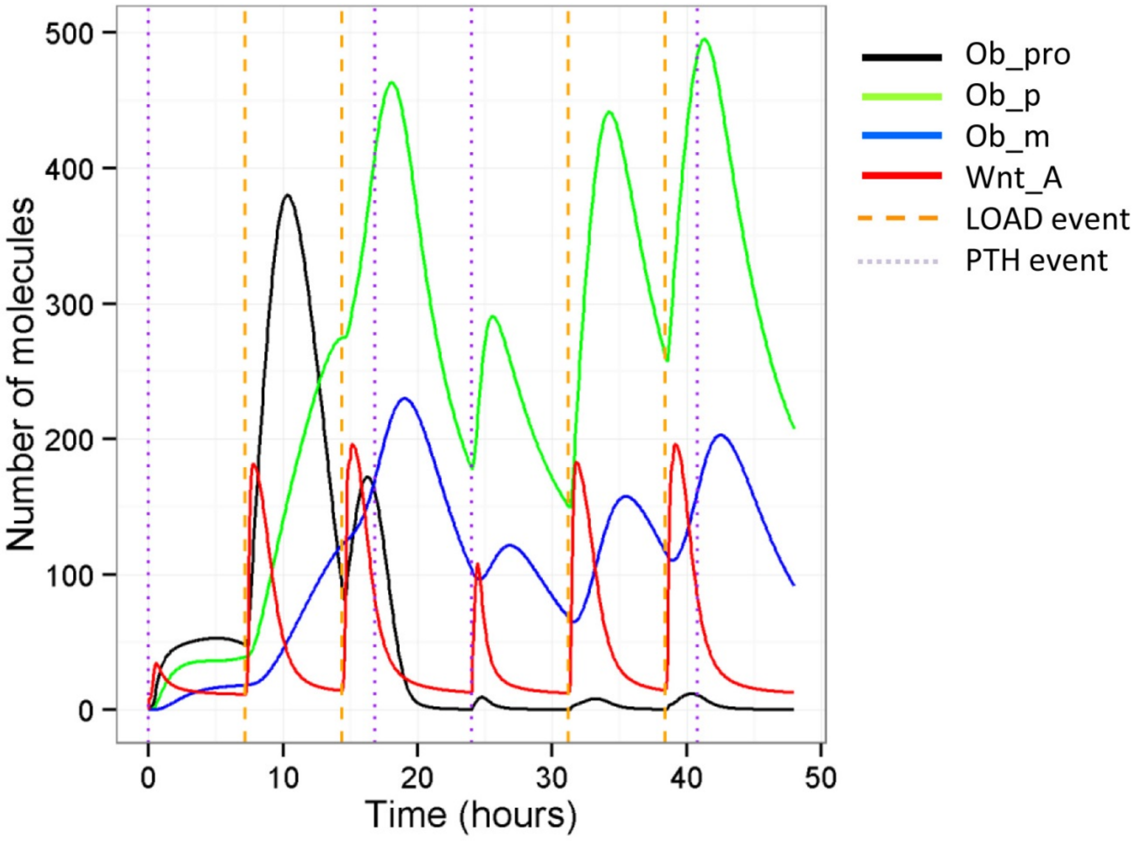

Supplementary Figure 4 Model output of sub-model "Osteoclast differentiation". The peak of PTH at the beginning of the simulation leads to an increase in secretion of MSCF and RANKL and both are increased further after the loading event at about 8 hours. This leads differentiation of HSCs into osteoclast precursors (Ocl_p), followed by differentiation into mature osteoclasts (Ocl_m). OPG levels start to increase as mature osteoblasts are formed, leading to less osteoclast differentiation and numbers of Ocl_m decline due to apoptosis. Simulations were performed over 2 day period to give a clearer picture of the dynamics.

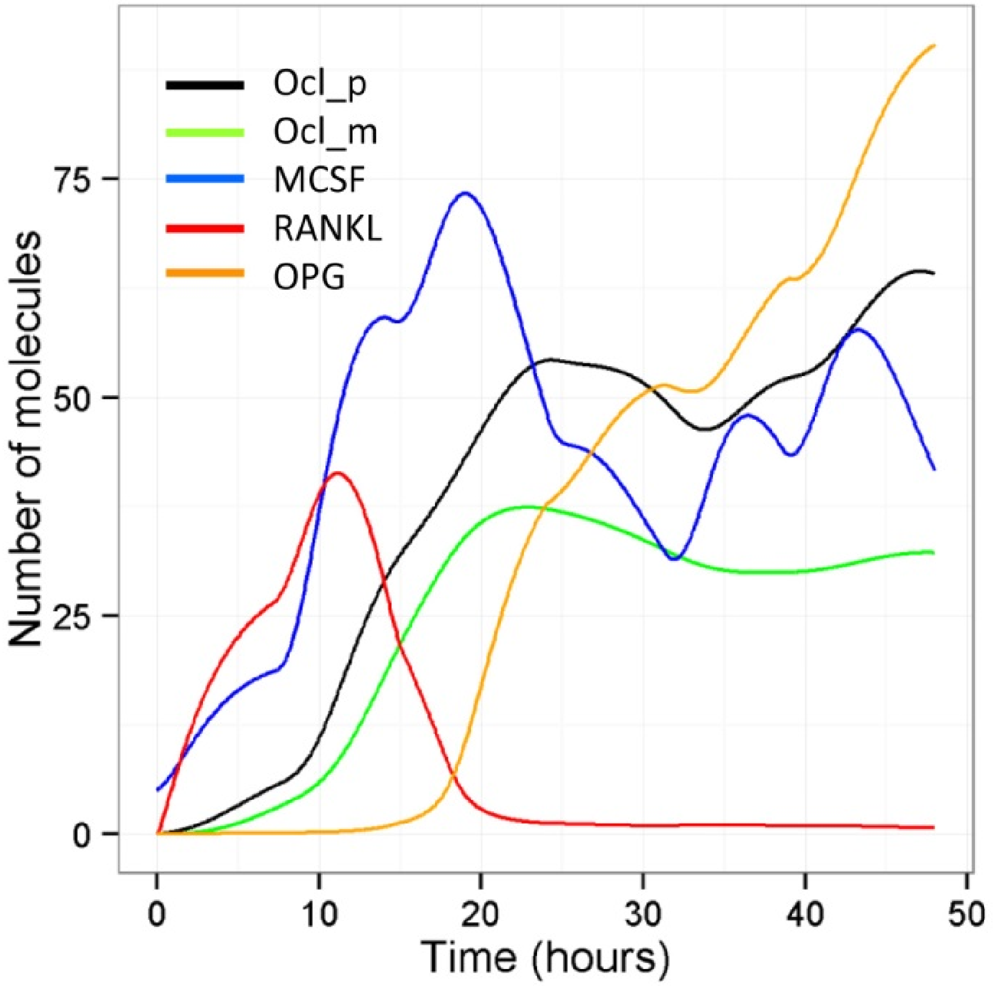

Supplementary Figure 5 Model output of sub-model "Tgfb". When mature osteoblasts (Ob_m) increase after loading or a peak in PTH levels, levels of secreted (inactive) Tgfb (Tgfb_I) increase, so that peaks of Tgfb_I correspond with peaks of Ob_m. Tgfb is activated (Tgfb_A) by mature osteoclasts (Ocl_m) and so peaks of Tgfb_A correspond to peaks of Ocl_m. Tgbf_I is scaled for easier visualisation. Simulations were performed over 10 day period to show how oscillations of bone cells affect Tgfb levels. Note that Tgfb_A is total pool including that which is bound to Ob_p.

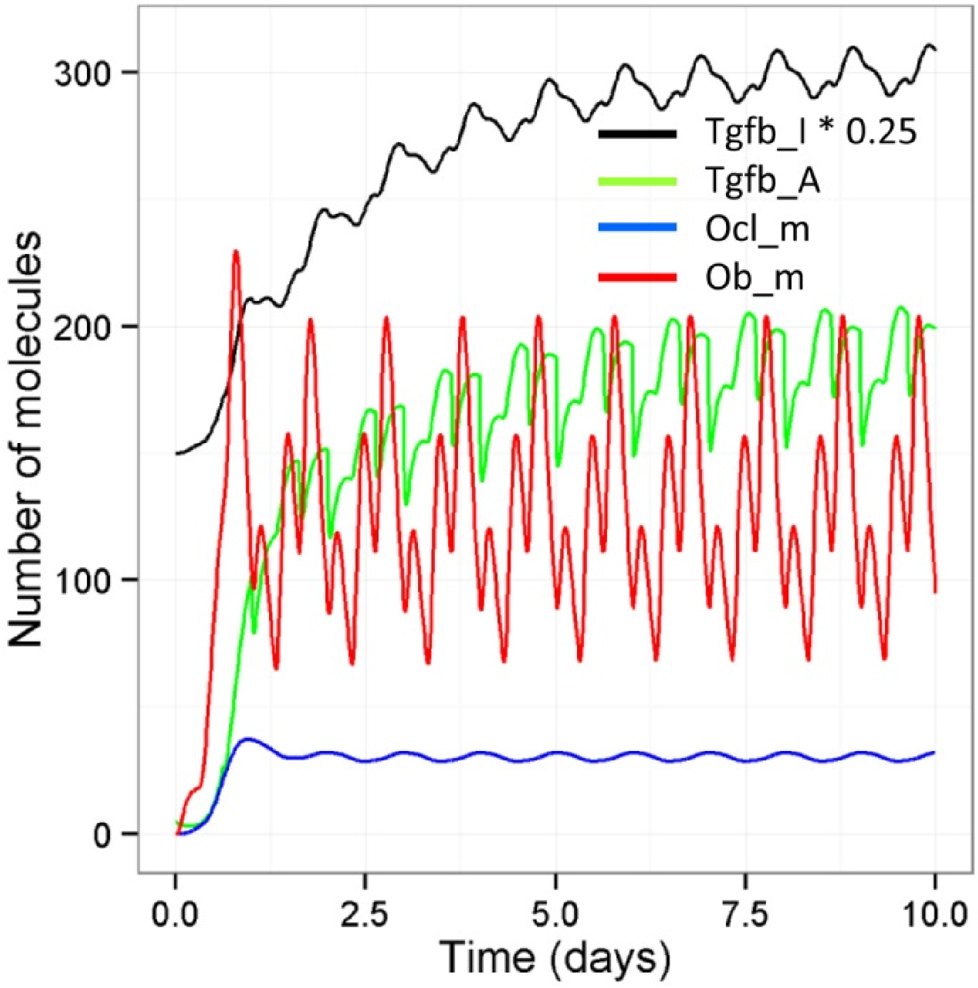

Supplementary Figure 6 Model output of sub-model "Bone turnover". During the time course, mature osteoblasts (Ob_m) and mature osteoclasts (Ocl_m) peak at different times so that the Ob_m/Ocl_m ratio changes with time. Bone mass increase when the ratio is high and declines when the ratio is decreased.

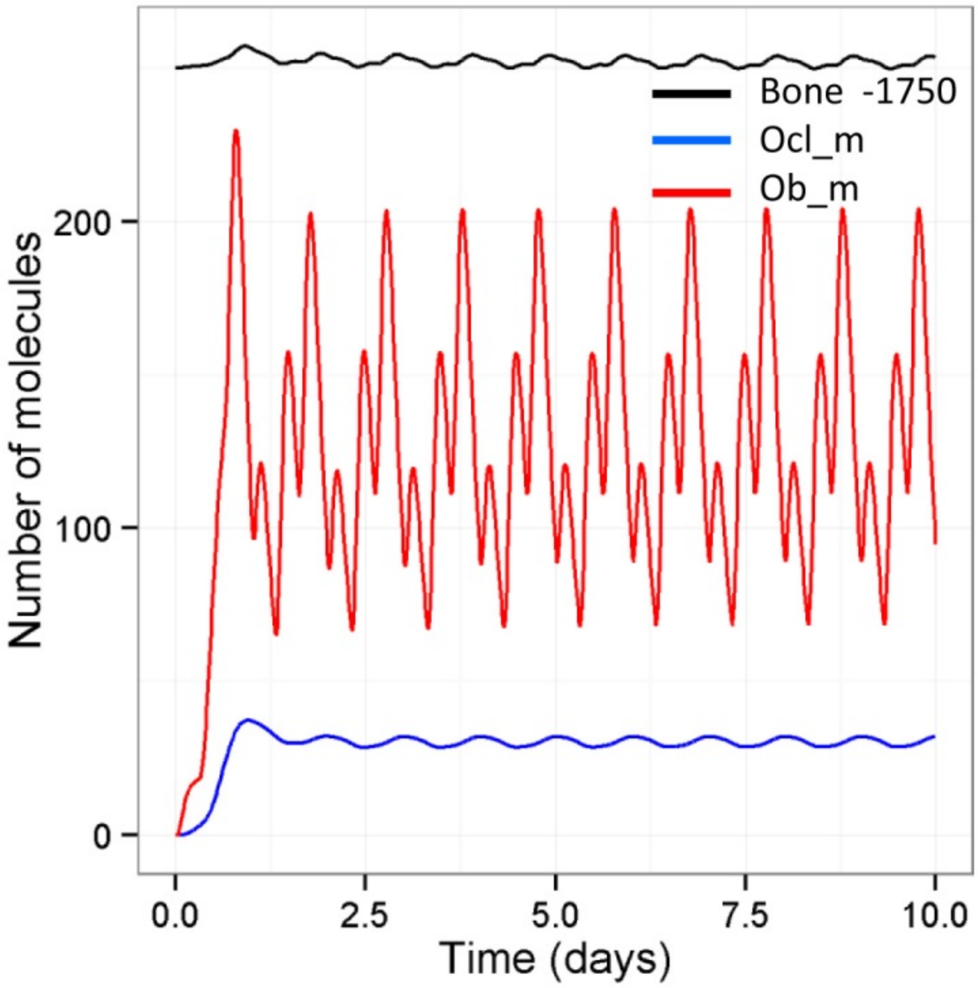

Supplementary Table 1. Details of model reactions and parameter values

Reaction ID	Reactants and products	Kinetic rate law
Unloading	LOAD → Sink	k_{unload} * LOAD
Osteocyte_activation	Ocy_I + LOAD → Ocy_A + LOAD	k_{actOcy} * Ocy_I * LOAD
Osteocyte_inactivation	Ocy_A → Ocy_I	$k_{inactOcy}$ * Ocy_A
Ocy_I_binding_by_PTH	Ocy_I+PTH→ Ocy_I_PTH	$k_{binOcyPTH}$ * Ocy_I * (PTH^2/(100^2+PTH^2))
Ocy_I_PTH_release	Ocy_I_PTH → Ocy_I +PTH	$k_{relOcyPTH}$ * Ocy_I_PTH
Osteocyte_activation_by_PTH	Ocy_I_PTH → Ocy_A+PTH	$k_{actOcyPth}$ * Ocy_I_PTH
Ocy_apoptosis	Ocy_I → Sink	$k_{deathOcy}$ * Ocy_I
Sost_secretion	Ocy_I→ Ocy_I+Sost	$k_{secSost}$ * Ocy_I
Sost_degradation	Sost → Sink	$k_{degSost}$ * Sost
Wnt_activation	Wnt_I → Wnt_A	k_{actWnt} * Wnt_I
Wnt_activation_by_PTH	Wnt_I+Ob_m_PTH → Wnt_A+Ob_m_PTH	$k_{actWntPth}$ * Wnt_I * Ob_m_PTH
Wnt_inactivation_by_Sost	Wnt_A+Sost → Wnt_I +Sost	$k_{inactWnt}$ * Wnt_A * Sost^2/(50^2+Sost^2)
MSC_differentiation_to_Ob_pro	MSC+Wnt_A → MSC+Wnt_A+Ob_pro	$k_{diffMSC}$ * MSC * Wnt_A^2/(50^2+Wnt_A^2)
Osteoblast_progenitor_ differentiation_by_Tgfb	Ob_pro+Tgfb_A → Ob_p+Tgfb_A	$k_{diffObproTgfb}$ * Ob_pro * Tgfb_A^2/(50^2+Tgfb_A^2)
Ob_precursor_differentiation	Ob_p → Ob_m	$k_{diffObP}$ * Ob_p
Ob_p_binding_by_PTH	Ob_p+PTH → Ob_p_PTH	$k_{binObpPTH}$ * Ob_p * (PTH^2/(100^2+PTH^2))
Ob_p_PTH_release	Ob_p_PTH → Ob_p+PTH	$k_{relObpPTH}$ * Ob_p_PTH
Ob_p_Tgfb_binding	Ob_p+Tgfb_A → Ob_p_Tgfb_A	$k_{binObpTgfb}$ * Ob_p * Tgfb_A
Ob_p_Tgfb_release	Ob_p_Tgfb_A → Ob_p+Tgfb_A	$k_{relObpTgfb}$ * Ob_p_Tgfb_A
Ob_maturation_to_Ocy	Ob_m → Ocy_I	k_{matOb} * Ob_m
Ob_maturation_to_Ocy_by_Tgfb	Ob_m+Tgfb_A → Ocy_I+Tgfb_A	$k_{matObTgfb}$ * Ob_m * Tgfb_A^2/(50^2+Tgfb_A^2)
Ob_m_bound_by_PTH	Ob_m+PTH → Ob_m_PTH	$k_{binObmPTH}$ * Ob_m * (PTH^2/(100^2+PTH^2))
Ob_m_PTH_release	Ob_m_PTH → Ob_m+PTH	$k_{relObmPTH}$ * Ob_m_PTH
Ob_m_apoptosis	Ob_m+Bax → Bax	$k_{deathOb}$ * Ob_m * Bax^2/(50^2+Bax^2)
Ob_m_PTH_apoptosis	Ob_m_PTH+Bax → Bax+PTH	$k_{deathOb}$ * Ob_m_PTH * Bax^2/(50^2+Bax^2)
HSC_differentiation_to_Ocl_p	HSC+MCSF → HSC+MCSF+Ocl_p	$k_{diffHSC}$ * HSC * $MCSF^2/(50^2$+MCSF^2)
Ocl_p_RANKL_binding	RANKL+Ocl_p → Ocl_p_RANKL	$k_{binOclpRANKL}$ * Ocl_p* RANKL
Ocl_p_RANKL_release	Ocl_p_RANKL → Ocl_p + RANKL	$k_{relOclpRANKL}$ * Ocl_p_RANKL
Osteoclast_precursor_ differentiation	Ocl_p_RANKL → Ocl_m	$k_{diffOclP}$ * Ocl_p_RANKL
Ocl_p_apoptosis	Ocl_p → Sink	$k_{deathOclp}$ * Ocl_p
RANKL_inhibition	OPG+RANKL → OPG_RANKL	$k_{inhibRANKL}$ * OPG * RANKL
OPG_RANKL_dissociation	OPG_RANKL → OPG+RANKL	$k_{relRANKL}$ * OPG_RANKL
OPG_RANKL_degradation	OPG_RANKL → Sink	$k_{degOPGRANKL}$ * OPG_RANKL
Osteoclast_apoptosis	Ocl_m → Sink	$k_{deathOcl}$ * Ocl_m
RANKL_degradation	RANKL → Sink	$k_{degRANKL}$ * RANKL
OPG_degradation	OPG → Sink	k_{degOPG} * OPG
MCSF_secretion_by_MSC	MSC → MSC+MCSF	$k_{secMCSFbyMSC}$ * MSC
MCSF_secretion_by_Ob_pro	Ob_pro → Ob_pro+MCSF	$k_{secMCSFbyObpro}$ * Ob_pro
MCSF_secretion_by_Ob_p	Ob_p → Ob_p+MCSF	$k_{secMCSFbyObp}$ * Ob_p
MCSF_secretion_by_Ob_p_PTH	Ob_p_PTH → Ob_p_PTH+MCSF	$k_{secMCSFbyObp}$ * Ob_p_PTH
MCSF_secretion_by_Ob_m	Ob_m → Ob_m+MCSF	$k_{secMCSFbyObm}$ * Ob_m
MCSF_secretion_by_Ob_m_PTH	Ob_m_PTH → Ob_m_PTH+MCSF	$k_{secMCSFbyObm}$ * Ob_m_PTH
MCSF_degradation	MCSF → Sink	$k_{degMCSF}$ * MCSF

OPG_secretion_by_Ob_p	Ob_p → Ob_p+OPG	$k_{secOPGbyObp}$ * Ob_p
OPG_secretion_by_Ob_p_PTH	Ob_p_PTH → Ob_p_PTH+OPG	$k_{secOPGbyObpPTH}$ * Ob_p_PTH
OPG_secretion_by_Ob_m	Ob_m → Ob_m+OPG	$k_{secOPGbyObm}$ * Ob_m
RANKL_secretion_by_Ocy_A	Ocy_A → Ocy_A+RANKL	$k_{secRANKLbyOcy}$ * Ocy_A
RANKL_secretion_by_Ocy_I	Ocy_I → Ocy_I+RANKL	$k_{secRANKLbyOcyI}$ * Ocy_I
RANKL_secretion_by_MSCs	MSC → MSC+RANKL	$k_{secRANKLbyMSC}$ * MSC
RANKL_secretion_by_Ob_p	Ob_p → Ob_p+RANKL	$k_{secRANKLbyObp}$ * Ob_p
RANKL_secretion_by_Ob_p_Tgfb_A	Ob_p_Tgfb_A → Ob_p_Tgfb_A+RANKL	$k_{secRANKLbyObpTgfb}$ * Ob_p_Tgfb_A
RANKL_secretion_by_Ob_p_PTH	Ob_p_PTH → Ob_p_PTH+RANKL	$k_{secRANKLbyObpPTH}$ * Ob_p_PTH
RANKL_secretion_by_Ob_pro	Ob_pro → Ob_pro+RANKL	$k_{secRANKLbyObpro}$ * Ob_pro
RANKL_secretion_by_Ob_m	Ob_m → Ob_m+RANKL	$k_{secRANKLbyObm}$ * Ob_m
RANKL_secretion_by_Ob_m_PTH_ enhanced	Ob_m_PTH → Ob_m_PTH+RANKL	$k_{secRANKLbyObmPTH}$ * Ob_m_PTH
Tgfb_secretion_by_Obm	Ob_m → Ob_m+Tgfb_I	$k_{secTgfb}$ * Ob_m
Tgfb_activation	Tgfb_I+Ocl_m → Tgfb_A+Ocl_m	$k_{actTgfb}$ * Tgfb_I * Ocl_m
Tgfb_degradation	Tgfb_A → Sink	$k_{degTgfb}$ * Tgfb_A
Tgfb_degradation_by_PTH	Tgfb_A+Ob_m_PTH → Ob_m_PTH	$k_{degTgfbPTH}$ * Tgfb_A * Ob_m_PTH
PTH_production	Source → PTH	k_{synPTH} * Source
PTH_degradation	PTH → Sink	k_{degPTH} * PTH
CREB_activation	Ob_m_PTH+CREB → Ob_m_PTH+CREB_P	$k_{actCreb}$ * CREB*Ob_m_PTH^2/ (100^2+Ob_m_PTH^2)
CREB_inactivation	CREB_P → CREB	$k_{inactCreb}$ * CREB_P
CREB_Runx2_binding	CREB_P+Runx2 → CREB_Runx2	$k_{binCrebRunx2}$ * CREB_P * Runx2
CREB_Runx2_release	CREB_Runx2 → CREB_P+Runx2	$k_{relCrebRunx2}$ * CREB_Runx2
Bcl2_synthesis	CREB_Runx2 → CREB_Runx2+Bcl2	$k_{synBcl2}$ * CREB_Runx2
Bcl2_degradation	Bcl2 → Sink	$k_{degBcl2}$ * Bcl2
Bax_Bcl2_binding	Bax+Bcl2 → Bax_Bcl2	$k_{binBaxBcl2}$ * Bax * Bcl2
Bax_Bcl2_release	Bax_Bcl2 → Bax+Bcl2	$k_{relBaxBcl2}$ * Bax_Bcl2
Runx2_degradation_via_PTH	Ob_m_PTH+Runx2 → Ob_m_PTH	$k_{degRunx2PTH}$ * Runx2 * Ob_m_PTH
Runx2_degradation	Runx2 → Sink	$k_{degRunx2}$ * Runx2
Runx2_synthesis	Source → Runx2	$k_{synRunx2}$ * Source
Bone_formation	Ob_m → Ob_m+Bone	$k_{formBone}$ * Ob_m
Bone_formation_Obm_PTH	Ob_m_PTH → Ob_m_PTH+Bone	$k_{formBone}$ * Ob_m_PTH
Bone_degradation	Ocl_m+Bone → Ocl_m	$k_{degBone}$ * Ocl_m * Bone

The ODE of each species in the model can be constructed from the reactions in which the species is a reactant or a product. If the species is a reactant, then the result of the reaction is that the species declines by one and so the term is negative and rate is given by the kinetic rate law. If the species is a product, then the result of the reaction is that the species increases by one and so the term is positive and the rate is given by the kinetic rate law. For example, OPG_RANKL is a product in one reaction: RANKL_inhibition, and a reactant in two reactions: OPG_RANKL_dissociation and OPG_RANKL_degradation. Therefore the ODE is:

$$\frac{d[OPG\_RANKL]}{dt} = k_{inhibRANKL}[OPG][RANKL] - k_{relRANKL}[OPG\_RANKL] - k_{degOPGRANKL}[OPG\_RANKL]$$

All the ODEs can be viewed in the COPASI file (Supplementary file 3).

Supplementary Table 2. Detail of model parameters

Parameter id	Value[a]	Comment
$k_{actCreb}$	9.0E-3 s^{-1}	CREB is activated rapidly (within a few minutes) after PTH binds to mature osteoblasts (Zhang et al., 2011).
k_{actOcy}	4.0E-3 $mol^{-1}s^{-1}$	Activation occurs within 1 hour of loading (Lara-Castillo et al., 2015)
$k_{actOcyPth}$	0.08 s^{-1}	Osteocytes are rapidly activated after PTH binding
$k_{actTgfb}$	2.0E-7 $mol^{-1}s^{-1}$	Tgfb activation by osteoclasts occurs
k_{actWnt}	0.03 s^{-1}	Activation occurs within 1 hour of loading (Lara-Castillo et al., 2015). This is due to downregulation of Sost
$k_{actWntPth}$	1.0E-3 s^{-1}	PTH binding to mature osteoblasts activates Wnt but at a lower rate than that induced by loading.
$k_{binBaxBcl2}$	0.01 $mol^{-1}s^{-1}$	Assume rapid kinetics of binding/dissociation of Bax/Bcl2 binding
$k_{binCrebRunx2}$	0.01 $mol^{-1}s^{-1}$	Assume rapid kinetics of binding/dissociation of CREB/Runx2 binding
$k_{binObmPTH}$	0.02 s^{-1}	PTH binds strongly to receptors on mature osteoblasts
$k_{binObpPTH}$	3.0E-4 s^{-1}	PTH binds to receptors on osteoblast precursors with less affinity than mature cells
$k_{binObpTgfb}$	2.0E-4 $mol^{-1}s^{-1}$	Tgfb binds to receptors on osteoblast precursors with fast kinetics compared to activation of Tgfb by osteoclasts (Lemaire et al., 2004)
$k_{binOclpRANKL}$	1.0E-3 $mol^{-1}s^{-1}$	Assume fast kinetics of binding/dissociation of RANKL/RANK receptors on osteoclast precursors
$k_{binOcyPTH}$	8.0E-3 s^{-1}	PTH binds to receptors on osteocytes with less affinity than mature osteoblasts
$k_{deathOb}$	2.4E-4 s^{-1}	Assumed a half-life of about two hours due to simplifying assumptions regarding spatial aspects.
$k_{deathOcl}$	6.5E-5 s^{-1}	Assumed a half-life of about three hours due to simplifying assumptions regarding spatial aspects.
$k_{deathOclp}$	1.0E-5 s^{-1}	Assumed a half-life of about one day due to simplifying assumptions regarding spatial aspects.
$k_{deathOcy}$	1.0E-8 s^{-1}	Half-life is about 25 years (Knothe Tate et al., 2004). This was speeded up about 10 fold for the model due to simplifying assumptions regarding spatial aspects.
$k_{degBcl2}$	2.5E-3 s^{-1}	Assumed that only free pools of Bcl2 are degraded with a half-life of about 5 minutes.
$k_{degBone}$	6.5E-9 $mol^{-1}s^{-1}$	10% of bone is remodeled in one year. This was speeded up about two-fold to allow shorter simulation times.
$k_{degMCSF}$	1.0E-4 s^{-1}	Half-life is about 2 hours (Rubin et al., 1998)
k_{degOPG}	4.0E-6 s^{-1}	Half-life is about 2 days (Lemaire et al., 2004)
$k_{degOPGRANKL}$	1.0E-5 s^{-1}	Assumed OPG and RANKL are both degraded when they are bound (Tat et al., 2006)
k_{degPTH}	2.0E-3 s^{-1}	Half-life is about 5 minutes (Bieglmayer et al., 2002).
$k_{degRANKL}$	3.0E-5 s^{-1}	Half-life of about 6 hours
$k_{degRunx2}$	1.0E-4 s^{-1}	Half-life is about 2 hours.
$k_{degRunx2PTH}$	3.0E-3 s^{-1}	PTH increases Runx2 degradation rate.
$k_{degSost}$	4.0E-3 s^{-1}	Half-life is about 3 minutes.

$k_{degTgfb}$	5.0E-5 s^{-1}	Half-life is about 4 hours.
$k_{degTgfbPTH}$	1.7E-5 $mol^{-1}s^{-1}$	PTH signaling leads to further Tgfb degradation.
$k_{diffHSC}$	5.5E-5 s^{-1}	Osteoclast precursors increase rapidly after loading and secretion of MCSF, peaking at about 10-15 hours post-loading.
$k_{diffMSC}$	6.5E-4 s^{-1}	Osteoblast pro-genitor cells increase rapidly after loading or PTH-induced activation of Wnt and peaks at about 3 hours after Wnt.
$k_{diffObP}$	1.0E-4 s^{-1}	Mature osteoblasts increase about 10 hours after the precursor cells.
$k_{diffObproTgfb}$	0.05 s^{-1}	Osteoblast precursors increase rapidly after Tgfb activation, peaking about 10 hours after the peak in progenitor cells
$k_{diffOclP}$	8.0E-5 s^{-1}	Mature osteoclasts increase after RANKL binds to osteoclast precursors.
$k_{formBone}$	3.07E-6 s^{-1}	Rate of formation was set to balance rate of degradation under normal loading and an intact PTH circadian rhythm.
$k_{inactCreb}$	1.0E-4 s^{-1}	Dephosphorylation of CREB has much slower kinetics than phosphorylation (time scale of hours) (Zhang et al., 2011).
$k_{inactOcy}$	2.0E-5 s^{-1}	Osteocyte activation by loading or PTH is transient so assume a gradual return to an inactive state with half-life of about 10 h in the absence of any further stimuli. .
$k_{inactWnt}$	0.8 s^{-1}	Wnt is rapidly inactivated in the presence of Sost.
$k_{inhibRANKL}$	1.0E-3 $mol^{-1}s^{-1}$	This is the rate at which OPG binds to RANKL to inhibit its activity. Assumed fast kinetics of binding/dissociation of OPG/RANKL binding.
k_{matOb}	2.0E-9 s^{-1}	A low proportion of osteoblast mature into osteocytes. This rate was adjusted so that pool of osteocytes was maintained.
$k_{matObTgfb}$	1.0E-8 s^{-1}	Tgfb increases the rate of osteoblast maturation
$k_{relBaxBcl2}$	0.5 s^{-1}	Assume rapid kinetics of binding/dissociation of Bax/Bcl2 binding
$k_{relCrebRunx2}$	0.01 s^{-1}	Assume rapid kinetics of binding/dissociation of CREB/Runx2 binding
$k_{relObmPTH}$	5.0E-3 s^{-1}	Assume rapid kinetics of binding/dissociation of PTH with mature osteoblasts.
$k_{relObpPTH}$	5.0E-3 s^{-1}	Assume rapid kinetics of binding/dissociation of PTH with osteoblast precursors.
$k_{relObpTgfb}$	0.01 s^{-1}	Assume rapid kinetics of binding/dissociation of Tgfb with osteoblast precursors.
$k_{relOclpRANKL}$	1.0E-3 s^{-1}	Assume rapid kinetics of binding/dissociation of RANKL with osteoclast precursors.
$k_{relOcyPTH}$	5.0E-3 s^{-1}	Assume rapid kinetics of binding/dissociation of PTH with osteocytes
$k_{relRANKL}$	1.0E-3 s^{-1}	Assumed rapid kinetics of binding/dissociation of OPG/RANKL binding.
$k_{secMCSFbyMSC}$	1.0E-5 s^{-1}	Rate of secretion was set to balance degradation rate.

$k_{secMCSFbyObm}$	1.0E-5 s^{-1}	Assumed different types of bone cell secreted MCSF at same rate.
$k_{secMCSFbyObm}$	1.0E-5 s^{-1}	
$k_{secMCSFbyObp}$	1.0E-5 s^{-1}	
$k_{secMCSFbyObp}$	1.0E-5 s^{-1}	
$k_{secMCSFbyObpro}$	1.0E-5 s^{-1}	
$k_{secOPGbyObm}$	1.0E-5 s^{-1}	Rate of secretion was set to balance degradation rate.
$k_{secOPGbyObp}$	2.0E-6 s^{-1}	Assumed less secretion of OPG by osteoblast precursor cells than mature cells.
$k_{secOPGbyObpPTH}$	1.0E-6 s^{-1}	Assumed PTH inhibited secretion of OPG.
$k_{secRANKLbyMSC}$	1.0E-6 s^{-1}	Since there is a constant pool of MSC, there is a low basal rate of RANKL secretion which is set to balance degradation rate giving a low basal level of about 2-3 molecules.
$k_{secRANKLbyObm}$	1.0E-7 s^{-1}	Assume mature osteoblasts secrete low levels of RANKL
$k_{secRANKLbyObmPTH}$	1.0E-6 s^{-1}	Assume a ten-fold increase in the rate of secretion of RANKL by mature osteoblasts when bound by PTH.
$k_{secRANKLbyObp}$	3.0E-6 s^{-1}	Assume osteoblast precursors secrete more RANKL than mature cells
$k_{secRANKLbyObpPTH}$	2.0E-5 s^{-1}	Assume an order of magnitude increase in the rate of secretion of RANKL by osteoblast precursors when bound by PTH.
$k_{secRANKLbyObpro}$	7.0E-6 s^{-1}	Assume osteoblast progenitor cells secrete more RANKL than unstimulated precursor cells but less than precursor cells bound by PTH.
$k_{secRANKLbyObpTgfb}$	4.0E-6 s^{-1}	Assume Tgfb increases the rate of RANKL secretion by osteoblast precursors but not to the same extent as PTH.
$k_{secRANKLbyOcy}$	1.0E-6 s^{-1}	Assume active osteocytes secrete RANKL at 10x the rate for inactive osteocytes.
$k_{secRANKLbyOcyI}$	1.0E-7 s^{-1}	Assume inactive osteocytes secrete low levels of RANKL.
$k_{secSost}$	7.5E-4 s^{-1}	Parameter set to maintain basal levels of Sost in the absence of loading or PTH peaks.
$k_{secTgfb}$	5.0E-5 s^{-1}	Parameter set to maintain basal levels of Tgfb.
$k_{synBcl2}$	5.0E-3 s^{-1}	Parameter set to maintain basal levels of Bcl2
k_{synPTH}	0.02 mol s^{-1}	Parameter set to maintain basal levels of PTH
$k_{synRunx2}$	5.0E-3 mol s^{-1}	Parameter set to maintain basal levels of Runx2
k_{unload}	3.5E-4 s^{-1}	Period of loading is about 30 minutes

[a]mol=number of molecules

Supplementary Table 3. Detail of model events for loading and PTH circadian rhythm

**(A) Model with 2 loads and 2 PTH peaks per day**		
**Event ID**	**Trigger**	**Assignment**
AddLoad1	X>300	LOAD=1
AddLoad2	X>600	LOAD=1
AddPTH1	X>700	PTH=150
AddPTH2	X>1000	PTH=170; X=0
**(B) Model with 1 load and 2 PTH peaks per day**		
AddLoad1	X>300	LOAD=1
AddPTH1	X>700	PTH=150
AddPTH2	X>1000	PTH=170; X=0
**(C) Model with 2 loads and 1 PTH peak per day**		
AddLoad1	X>300	LOAD=1
AddLoad2	X>600	LOAD=1
AddPTH1	X>1000	PTH=150; X=0
**(D) Model with 1 load and 1 PTH peak per day**		
AddLoad1	X>300	LOAD=1
AddPTH1	X>1000	PTH=150; X=0

Supplementary Table 4. Results of sensitivity analysis

Parameters with positive effects on bone mass		Parameters with negative effects on bone ,mass	
kformBone	0.818	krelObmPTH	-0.071
kdeathOcl	0.816	kdiffOclP	-0.073
kdegMCSF	0.489	kdegOPG	-0.074
kdiffObP	0.425	krelRANKL	-0.074
kdiffMSC	0.378	kbinOclpRANKL	-0.078
kdegSost	0.281	kinactCreb	-0.085
ksynPTH	0.232	kdegRunx2PTH	-0.108
kactWnt	0.169	krelCrebRunx2	-0.109
ksecOPGbyObm	0.140	ksecMCSFbyMSC	-0.111
ksynBcl2	0.123	ksecTgfb	-0.111
kbinBaxBcl2	0.123	kbinObpTgfb	-0.112
ksynRunx2	0.114	ksecRANKLbyObpTgfb	-0.113
krelObpTgfb	0.112	ksecRANKLbyObp	-0.122
kbinCrebRunx2	0.109	kdegBcl2	-0.123
kbinOcyPTH	0.088	krelBaxBcl2	-0.123
kdeathOclp	0.078	ksecMCSFbyObm	-0.140
kinhibRANKL	0.075	kunload	-0.178
kdegOPGRANKL	0.074	ksynX	-0.193
kbinObmPTH	0.073	kinactWnt	-0.201
kdegTgfbPTH	0.073	kinactOcy	-0.226
krelOclpRANKL	0.073	ksecMCSFbyObp	-0.231
kactOcy	0.072	ksecSost	-0.278
kactCreb	0.051	kdegPTH	-0.290
		ksecRANKLbyOcy	-0.303
		kdiffHSC	-0.441
		kdeathOb	-0.802
		kdegBone	-0.815

Sensitivity analysis of the deterministic model with two loading and two PTH events per day (Table S3) was carried out in Copasi using subtask “Time series”. Parameters which positively or negatively affect bone mass by more than 5%. All sensitivity values are scaled. Green/red indicates parameters which increase/decrease bone mass if their value increases. Full results are shown in Supplementary File 2.

Supplementary Table 5. Results of parameter scans. Each parameter which had more than 10% effect on bone mass was varied from half to twice its initial value over ten intervals. The table shows the effect of doubling each parameter value on bone mass, mature osteoblasts and mature osteoclasts compared to default value. Parameters are listed in order of their sensitivities (as shown in Supplementary Table 4), note that in some cases, halving the parameter value had a greater effect on bone mass (e.g. $k_{deathOcl}$).

Parameter name	Bone mass (% change)	Ob_m (% change)	Ocl_m (% change)
kformBone	81.46	0.00	0.00
kdeathOcl	56.88	-0.01	-49.95
kdegMCSF	62.81	-0.07	-53.44
kdiffObP	26.22	-0.77	-27.30
kdiffMSC	38.00	114.77	52.07
kdegSost	23.75	74.86	38.00
ksynPTH	47.07	113.39	39.87
kactWnt	13.55	42.35	23.02
ksecOPGbyObm	12.30	0.01	-13.95
ksynBcl2	13.48	18.37	1.56
kbinBaxBcl2	13.48	18.40	1.55
ksynRunx2	13.48	18.37	1.56
krelObpTgfb	6.15	-0.01	-7.29
kbinCrebRunx2	10.71	14.66	1.23
kdegRunx2PTH	-5.41	-7.38	-0.71
krelCrebRunx2	-5.43	-7.42	-0.71
ksecMCSFbyMSC	-8.04	0.00	10.42
ksecTgfb	-9.45	-0.02	12.66
kbinObpTgfb	-9.23	-0.03	12.36
ksecRANKLbyObpTgfb	-9.33	-0.01	12.56
ksecRANKLbyObp	-9.87	-0.02	13.34
kdegBcl2	-5.79	-7.89	-0.77
krelBaxBcl2	-5.79	-7.89	-0.77
ksecMCSFbyObm	-9.38	0.00	12.32
kunload	-8.49	-25.40	-15.80
ksynX	29.63	84.08	36.76
kinactWnt	-11.17	-34.09	-21.82
kinactOcy	-11.50	-41.73	-29.91
ksecMCSFbyObp	-12.50	-0.01	16.91
ksecSost	-13.59	-44.85	-30.78
kdegPTH	-6.30	-22.64	-15.29
ksecRANKLbyOcy	-14.94	-0.03	21.20
kdiffHSC	-19.70	-0.02	28.56
kdeathOb	-39.31	-53.91	-7.55
kdegBone	-48.24	0.00	0.00

The parameters which were shown to have more than 10% effect on bone mass in the sensitivity analysis (see Supplementary File 2) were varied in the range 0.5*k*-2*k*, where *k* is the parameter value, and deterministic simulations were carried out over 10 intervals in COPASI. The table shows the effect of a 2-fold increase in each of the parameters on bone mass, levels of mature osteoblasts (Ob_m), and levels of mature osteoclasts (Ocl_m) averaged over the final 20 time-points (final 2 days of the simulation) because levels of Ob_m and Ocl_m fluctuated during each 24 h period. The deterministic model with loading (activated every 24 h by an event) and a circadian rhythm of PTH was used for this analysis.

References

BIEGLMAYER, C., PRAGER, G. & NIEDERLE, B. 2002. Kinetic analyses of parathyroid hormone clearance as measured by three rapid immunoassays during parathyroidectomy. *Clin Chem,* 48**,** 1731-8.

KNOTHE TATE, M. L., ADAMSON, J. R., TAMI, A. E. & BAUER, T. W. 2004. The osteocyte. *The International Journal of Biochemistry & Cell Biology,* 36**,** 1-8.

LARA-CASTILLO, N., KIM-WEROHA, N. A., KAMEL, M. A., JAVAHERI, B., ELLIES, D. L., KRUMLAUF, R. E., THIAGARAJAN, G. & JOHNSON, M. L. 2015. In vivo mechanical loading rapidly activates β-catenin signaling in osteocytes through a prostaglandin mediated mechanism. *Bone,* 76**,** 58-66.

LEMAIRE, V., TOBIN, F. L., GRELLER, L. D., CHO, C. R. & SUVA, L. J. 2004. Modeling the interactions between osteoblast and osteoclast activities in bone remodeling. *J Theor Biol,* 229**,** 293-309.

RUBIN, J., BISKOBING, D. M., JADHAV, L., FAN, D., NANES, M. S., PERKINS, S. & FAN, X. 1998. Dexamethasone promotes expression of membrane-bound macrophage colony-stimulating factor in murine osteoblast-like cells. *Endocrinology,* 139**,** 1006-12.

TAT, S. K., PADRINES, M., THEOLEYRE, S., COUILLAUD-BATTAGLIA, S., HEYMANN, D., REDINI, F. & FORTUN, Y. 2006. OPG/membranous--RANKL complex is internalized via the clathrin pathway before a lysosomal and a proteasomal degradation. *Bone,* 39**,** 706-15.

ZHANG, R., EDWARDS, J. R., KO, S. Y., DONG, S., LIU, H., OYAJOBI, B. O., PAPASIAN, C., DENG, H. W. & ZHAO, M. 2011. Transcriptional regulation of BMP2 expression by the PTH-CREB signaling pathway in osteoblasts. *PLoS One,* 6**,** e20780.
